# Updated insights into the molecular networks for NLRP3 inflammasome activation

**DOI:** 10.1038/s41423-025-01284-9

**Published:** 2025-04-30

**Authors:** Seungwha Paik, Jin Kyung Kim, Hyo Jung Shin, Eun-Jin Park, In Soo Kim, Eun-Kyeong Jo

**Affiliations:** 1 Department of Microbiology, Chungnam National University College of Medicine, Daejeon, Republic of Korea; 2 Department of Medical Science, Chungnam National University College of Medicine, Daejeon, Republic of Korea; 3 System Network Inflammation Control Research Center, Chungnam National University College of Medicine, Daejeon, Republic of Korea; 4 Biomedical Research Institute, Chungnam National University Hospital, Daejeon, Republic of Korea; 5 Department of Microbiology, Keimyung University School of Medicine, Daegu, Republic of Korea; 6 Department of Biochemistry and Cell Biology, Eulji University School of Medicine, Daejeon, Republic of Korea; 7 Brain Research Institute, Chungnam National University College of Medicine, Daejeon, Republic of Korea; 8 Department of Pharmacology, Chungnam National University College of Medicine, Daejeon, Republic of Korea

**Keywords:** NLRP3 inflammasome, Pyroptosis, Post-translational modification (PTM), Licensing, Spatiotemporal, Inflammatory disease, Inflammasome, NOD-like receptors, Infection, Immune cell death, Interleukins

## Abstract

Over the past decade, significant advances have been made in our understanding of how NACHT-, leucine-rich-repeat-, and pyrin domain-containing protein 3 (NLRP3) inflammasomes are activated. These findings provide detailed insights into the transcriptional and posttranslational regulatory processes, the structural–functional relationship of the activation processes, and the spatiotemporal dynamics of NLRP3 activation. Notably, the multifaceted mechanisms underlying the licensing of NLRP3 inflammasome activation constitute a focal point of intense research. Extensive research has revealed the interactions of NLRP3 and its inflammasome components with partner molecules in terms of positive and negative regulation. In this Review, we provide the current understanding of the complex molecular networks that play pivotal roles in regulating NLRP3 inflammasome priming, licensing and assembly. In addition, we highlight the intricate and interconnected mechanisms involved in the activation of the NLRP3 inflammasome and the associated regulatory pathways. Furthermore, we discuss recent advances in the development of therapeutic strategies targeting the NLRP3 inflammasome to identify potential therapeutics for NLRP3-associated inflammatory diseases. As research continues to uncover the intricacies of the molecular networks governing NLRP3 activation, novel approaches for therapeutic interventions against NLRP3-related pathologies are emerging.

## Introduction

Inflammasomes function as cytosolic protein complexes that regulate host immune responses to infectious microbes and damage stimuli [1]. The roles and mechanisms of the NACHT-, leucine-rich repeat- (LRR), and pyrin domain-containing protein 3 or nucleotide-binding oligomerization domain-like receptor (NLR) protein 3 (NLRP3) inflammasome have been well characterized in innate immune and inflammatory responses [2–6]. NLRP3 serves as a pivotal sensor within the inflammasome complex and is triggered by an array of stimuli, such as pathogen-associated molecules and damage-associated molecules [7]. Upon recognition of these signals, NLRP3 undergoes crucial transformation from an inactive homo-oligomeric form to an active multimeric inflammasome complex through several steps, i.e., priming, licensing, and assembly, leading to the processing of pro-caspase-1 and the maturation of interleukin-1β (IL-1β) and IL-18 [7]. Understanding how diverse stimuli converge into the common assembly of protein complexes involving the transition of NLRP3 from inactive to active states requires deciphering the mechanisms by which the delicately controlled NLRP3 inflammasome is achieved.

Central to the NLRP3 activation cascade is the assembly of NLRP3 with the adaptor molecule apoptosis-associated speck-like protein containing a caspase-recruitment domain (ASC), which forms helical oligomers, thereby facilitating the recruitment of pro-caspase-1 to the inflammasome complex [2, 8]. This assembly triggers the self-processing and activation of caspase-1, the effector of the NLRP3 inflammasome, leading to the cleavage of pro-IL-1β and pro-IL-18 and the secretion of mature IL-1β and IL-18, respectively, which are potent proinflammatory cytokines [9–11]. Caspase-1 activation can trigger the cleavage of gasdermin D (GSDMD), inducing pyroptosis—a form of programmed cell death characterized by cell membrane rupture and the release of proinflammatory contents [12]. The recent elucidation of the cryoelectron microscopic structure of NLRP3 has significantly advanced our understanding of the structural and functional characteristics of NLRP3 inflammasome activation [13]. The balanced activation of inflammatory cytokine secretion and pyroptotic cell death is crucial for host defense against pathogenic stimuli and harmful environmental factors; however, overactivation of the NLRP3 inflammasome is related to the pathogenesis of inflammatory diseases, such as diabetes, cancer, and neurodegenerative disease [3, 5, 14]. Therefore, understanding how NLRP3 inflammasome activation is regulated is essential for developing strategies to target the pathways or molecules involved in NLRP3 inflammasome activation in different contexts.

In this Review, we provide a brief overview of the structures of the components of the NLRP3 inflammasome and complex assembly, followed by three essential steps in the executable model for understanding the mechanisms of NLRP3 inflammasome activation—priming, licensing, and assembly, which precede proinflammatory cytokine release and pyroptotic cell death. In addition, we provide a comprehensive analysis of the spatiotemporal and interactive molecular networks that coordinate NLRP3 inflammasome activation. Finally, we discuss the current development of potential therapeutics targeting NLRP3 inflammasome activation, including a diverse array of therapeutic candidates for various NLRP3-associated inflammatory diseases. These efforts provide insights into translational research that bridges basic mechanisms, thereby revealing the therapeutic potential of NLRP3 regulation to improve the clinical outcomes of patients with NLRP3-related diseases.

## Overview of NLRP3 inflammasome activation: classification and structure

### Classification of the NLRP3 inflammasome

Canonical, noncanonical, and alternative NLRP3 inflammasome pathways sense pathogens and danger signals to activate innate immune responses and pyroptosis [3]. Indeed, numerous pathogenic and danger stimuli can activate the canonical NLRP3 inflammasome, which is an intracellular multiprotein platform that can activate caspase-1, followed by the maturation of IL-1β, IL-18, and IL-37 and the induction of pyroptosis via the processing of GSDMD [2, 3, 15]. The noncanonical NLRP3 inflammasome can be activated by sensing cytoplasmic lipopolysaccharide (LPS), a process mediated by caspase-11 in mice (caspase-4 and caspase-5 in humans), leading to gasdermin D cleavage and pyroptosis [16]. Furthermore, the activation of alternative inflammasome pathways bypasses the priming step, and these pathways are activated by the engagement of TLR4 by extracellular LPS and TIR domain-containing adapter-inducing interferon-β (TRIF) and caspase-8, which are independent of K^+^ efflux, ASC speck formation, or pyroptosis. This pathway seems to be unique to the activation of the NLRP3 inflammasome in monocytes [17, 18]. Controlled activation of the NLRP3 inflammasome is required for host protective responses during pathogenic infections. Early studies reported that bacteria, viruses, parasites, and fungi can activate the NLRP3 inflammasome to elicit host protection [19]. According to a recent study, the polysaccharide galactosaminogalactan of *Aspergillus fumigatus* triggers NLRP3 inflammasome activation by interacting with ribosomal proteins, thereby promoting innate immune responses [20]. Nonetheless, uncontrolled inflammasome activation may lead to detrimental responses in the context of infections and other NLRP3-associated diseases.

For a considerable period, the activation of the canonical NLRP3 inflammasome complex is believed to require two signals—priming (signal 1) and assembly (signal 2). Recent advances have underscored the significant role and intricate mechanisms of posttranslational modifications (PTMs) on the components of the NLRP3 inflammasome in the licensing step for inflammasome activation [21, 22]. Additionally, significant efforts to identify the binding partners of NLRP3, such as the NIMA-related kinase 7 (NEK7)-NLRP3 interaction [23], have greatly enhanced our understanding of the molecular regulation of NLRP3 inflammasome activation. Numerous mechanisms, including ionic alterations, organelle homeostasis, and spatiotemporal dynamics, are believed to orchestrate NLRP3 inflammasome activation in different contexts involving NLRP3 stimuli, pathological conditions, and various cell and tissue types [3, 5, 7, 24, 25]. The updates of the molecular networks for NLRP3 inflammasome activation are discussed in detail in the following sections.

Noncanonical inflammasome activation is mediated by murine caspase-11 (human caspase-4 or caspase-5), which reacts to cytosolic LPS and leads to pore formation through GSDMD, resulting in pyroptosis [16]. Recent studies have shown that not only LPS but also bacterial mRNA, which indicates bacterial viability, is required for the noncanonical activation of the NLRP3 inflammasome in macrophages [26]. Unlike conventional NLRP3 activation, the alternative NLRP3 inflammasome pathway is activated only by toll-like receptor (TLR) agonists, such as LPS, and does not require K^+^ efflux in human monocytes [17]. The receptor-interacting serine/threonine-protein kinase 1 (RIPK1)–fas-associated protein with death domain (FADD)–caspase-8 signaling cascade was activated downstream of TLR4–TRIF signaling to activate NLRP3 upon LPS treatment, but ASC speck formation or pyroptosis in the classical NLRP3 pathway was not observed [17]. Furthermore, the short isoform of the cellular FLICE-like inhibitory protein, activated by nuclear factor (NF)-κB, inhibits alternative caspase-8-mediated NLRP3 inflammasome activation [27]. Notably, human monocytes can activate the NLRP3 inflammasome through K^+^ and Cl^−^ efflux without the need for a priming step [28]. Studies must evaluate diverse inflammasome responses, regulators, and sophisticated mechanisms that can revolutionize the new management of NLRP3-related infectious and inflammatory diseases.

### Structure of the NLRP3 inflammasome

Although primarily localized in the cytoplasm of immune cells, such as macrophages, monocytes, neutrophils, and T cells, the NLRP3 inflammasome has also been reported in epithelial cells [29, 30] and skeletal muscle cells [31]. The NLRP3 inflammasome comprises three essential components: a sensor (NLRP3), an adaptor (ASC), and an effector (caspase-1). NLRP3, which serves as a sensor of the NLRP3 inflammasome, is a member of the NLR protein family and features three distinct domains: (a) an N-terminal pyrin domain (PYD) that facilitates interactions with ASC [32]; (b) a NACHT domain consisting of a central nucleotide-binding domain (NBD), helical domain 1, winged helix domain, and helical domain 2, which is crucial for the self-association of NLRP3 [33]; and (c) a C-terminal LRR domain that is responsible for ligand sensing and autoinhibition by folding back onto the NBD [34]. Phylogenetic analyses suggest that NLRP genes are related to mammalian reproduction and are subject to evolutionary conservation [35]; however, the NLRP-ASC-caspase-1 inflammasome complexes have only been discovered in vertebrates [36]. Among the NLRP genes, NLRP3 is the most conserved. For example, NLRP3 is 82% identical between humans and mice; however, NLRP5 is only 50% identical [35]. Although NLRP3 is the most extensively studied mammalian NLR in the inflammasome, no direct NLRP3 orthologs have been discovered in fish [37, 38], implying that the evolution of NLRP3 in mammals is associated with their more complex immune regulation and inflammatory diseases, which are less common in fish. The adaptor ASC consists of a PYD at the N-terminus and a caspase-recruitment domain (CARD) at the C-terminus. Full-length caspase-1 comprises a CARD at the N-terminus, a large catalytic domain (p20) in the central region, and a small catalytic subunit domain (p10) at the C-terminus [8, 39]. The activation of caspase-1, which is synthesized as an inactive zymogen, is crucial for NLRP3 inflammasome activation. Its function involves converting pro-IL-1β and pro-IL-18 into their active forms through proteolytic activation. Caspase-1 contributes to the formation of GSDMD N-terminal fragment pores, which in turn trigger pyroptotic cell death [12]. Notably, inflammation and pyroptosis do not necessarily occur simultaneously [40]. A recent structural analysis-driven understanding of the confirmed changes in and assembly of the NLRP3 inflammasome via cryoelectron microscopy will be discussed later in the section on the spatiotemporal regulation of NLRP3 inflammasome activation.

## Priming (transcriptional activation) of the NLRP3 inflammasome

Among all NLRs, NLRP3 stands out for its characteristic; namely, basal expression alone is insufficient to activate the inflammasome in quiescent cells. Signal 1 (priming) acts as the preparatory stage for the subsequent assembly phase of NLRP3 inflammasome activation. Direct regulation of NLRP3 expression is crucial for this process [41]. A prototypical example of priming is the initiation of TLR4 activation by LPS. This event triggers the signaling cascade that activates NF-κB, leading to the upregulation of the expression of inactive NLRP3, pro-IL-1β, and pro-IL-18. FADD and caspase-8 play an accessory role in NF-κB-mediated NLRP3 transcription [42]. Additionally, TLR2 facilitates NLRP3 inflammasome activation through an early MyD88–IRAK1-dependent pathway, thus providing a priming signal [43]. The release of the danger signal high mobility group box 1 from microglia initiates NLRP3 inflammasome pathway priming through TLR2/TLR4 [44]. Retinol binding protein 4, the primary retinol carrier in serum, is elevated in individuals with obesity and type 2 diabetes and contributes to the priming of the NLRP3 inflammasome through TLR2 and TLR4 [45]. In addition to TLR ligands, IL-1β or tumor necrosis factor (TNF) serve as priming signals for NF-κB activation, resulting in increased expression of NLRP3 and pro-IL-1β [46, 47]. Furthermore, α-synuclein uptake regulates NLRP3 expression via the CD36/Fyn kinase/PKCδ pathway [48].

**Fig. 1:**
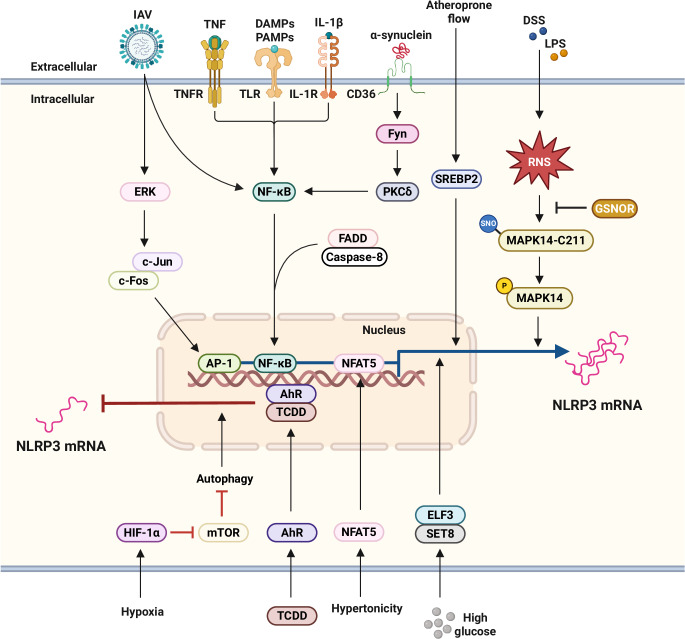
Transcriptional regulation of NLRP3 expression. The activation of TLRs by DAMPs, PAMPs, and pro-inflammatory cytokines such as TNF and IL-1β triggers NF-κB activation, which subsequently induces the transcription of the NLRP3 gene. This NF-κB-dependent transcription is mediated by FADD and caspase-8. During IAV infection, NLRP3 transcription is also upregulated via the ERK/c-Jun/AP-1 signaling pathway. In response to α-synuclein, Fyn kinase regulates PKCδ-mediated NF-κB activation, thereby promoting NLRP3 expression. Furthermore, atheroprone flow activates the NLRP3 inflammasome through SREBP2 activation. Under hypertonic stress, NFAT5 functions as a transcription factor to enhance NLRP3 expression. In endothelial cells exposed to high glucose conditions, ELF3 interacts with SET8 to regulate NLRP3 transcription. Conversely, during TCDD exposure, AhR suppresses NLRP3 expression. In models of LPS-induced septic shock and DSS-induced colitis, GSNOR modulates NLRP3 expression by reducing *S*-nitrosylated MAPK14 levels. Under hypoxic conditions, the accumulation of HIF-1α inhibits NLRP3 transcription by downregulating mTOR signaling and promoting autophagy, particularly in the context of DSS-induced colitis. AhR aryl hydrocarbon receptor, DAMP damage-associated molecular pattern, DSS dextran sodium sulfate, ELF3 transcription factor E74-like factor 3, FADD fas-associated death domain, GSNOR *S*-nitrosoglutathione reductase, HIF-1α hypoxia-inducible factor 1-alpha, IAV influenza A virus, LPS lipopolysaccharide, MAPK14 mitogen-activated protein kinase 14, mTOR mammalian target of rapamycin, NFAT5 nuclear factor of activated T cells 5, PAMP pattern-associated molecular pattern, PKCδ protein kinase Cδ, RNS reactive nitrogen species, SNO *S*-nitrosation, SREBP2 sterol regulatory element-binding protein2, TCDD 2,3,7,8-tetrachlorodibenzo-p-dioxin, TLR toll-like receptor, TNF tumor necrosis factor, TNFR tumor necrosis factor receptor

In addition to the NF-κB pathway, various signals and potential binding sites modulate NLRP3. For example, sterol regulatory element-binding protein 2 (SREBP2) regulate NLRP3 through lipid or glycolytic metabolism [49–51]. Indeed, NLRP3 is associated with SREBP cleavage-activating protein (SCAP)-SREBP2 and is translocated to the Golgi apparatus, where it is positioned close to a mitochondrial cluster for inflammasome assembly, thereby coordinating cholesterol metabolism with the inflammatory response specifically in macrophages [50]. Aryl hydrocarbon receptors, which are activated by pollutants, inhibit NLRP3 transcription by binding to its response element [52]. Metabolic stress prompts the induction of NLRP3 through nuclear factor of activated T cells 5 in the vascular endothelium, although hypoxia-induced hypoxia-inducible factor 1/2α and autophagy activation downregulates NLRP3 through mammalian target of rapamycin (mTOR) inhibition in colonic tissues [53, 54]. Recent studies have indicated that the activator protein-1 signaling pathway is involved in the transcriptional activation of pro-IL-1β in the priming step of NLRP3 inflammasome activation during influenza A virus infection [55]. In LPS-induced septic shock and dextran sodium sulfate (DSS)-induced colitis, *S*-nitrosoglutathione reductase regulates NLRP3 transcription through *S*-nitrosylation and the modulation of mitogen-activated protein kinase 14 activity [56]. Thus, multiple transcription factors are involved in NLRP3 priming, suggesting that different transcriptional programs are involved in inflammasome activation (Fig. 1). The identified transcription factors and pathways may contribute to maintaining the balance between activating and inhibitory signals. Future efforts will reveal positive and negative regulators and their detailed mechanisms of transcriptional activation of the NLRP3 inflammasome in different contexts and cells.

## The second signal of NLRP3 inflammasome activation

Next, we briefly discuss the updated mechanisms involved in the activation of the NLRP3 inflammasome complex (signal 2). The licensing step, which enables the PTMs of the NLRP3 inflammasome components required for activation, is examined in the following section.

**Fig. 2:**
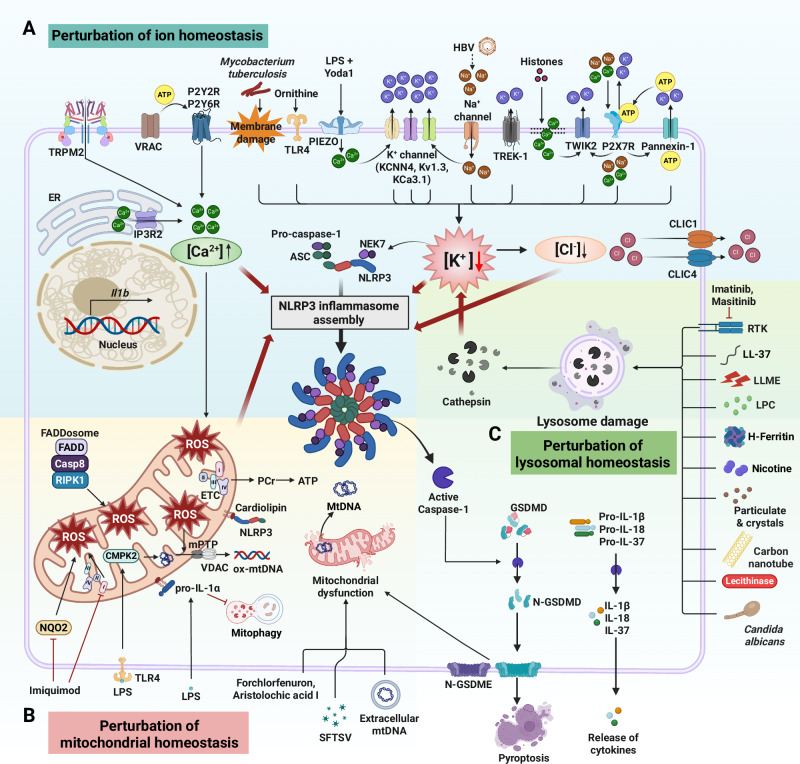
Second signal of NLRP3 inflammasome activation. **A** Perturbation of ion homeostasis perturbation. Membrane damage induced by *Mycobacterium tuberculosis* and TLR4 activation triggered by ornithine lead to K⁺ efflux. In addition, Ca²⁺ influx through the mechanosensitive channel PIEZO is converted into K⁺ efflux via KCNN4, thereby promoting NLRP3 inflammasome activation following stimulation with LPS and Yoda1. K⁺ efflux is further facilitated by various ion channels, including Kv1.3, KCa3.1, and TREK-1, ultimately contributing to NLRP3 activation. HBV activates the NLRP3 inflammasome through both K⁺ efflux and Na⁺ influx. Moreover, extracellular histones induce Ca²⁺ influx and recruit TWIK2 to the plasma membrane, resulting in K⁺ efflux through the TWIK2 channel. Extracellular ATP, released via pannexin-1 channels, binds to P2X7 receptors, leading to both Ca²⁺ influx and K⁺ efflux. Subsequently, K⁺ efflux promotes Cl⁻ efflux through CLIC1 and CLIC4, further contributing to NLRP3 inflammasome activation. Additionally, Ca²⁺ influx via TRPM2 induces mtROS production, providing an additional activation signal. VRAC activation, ATP release, and subsequent P2YR activation also participate in this process. Together, these ionic fluxes converge to promote NLRP3 inflammasome activation. **B** Perturbation of mitochondrial homeostasis. Extracellular mtDNA, SFTSV, forchlorfenuron, and aristolochic acid I have been shown to induce mitochondrial dysfunction. Gasdermin processing is essential for the release of mtDNA into the cytosol. Imiquimod inhibits mitochondrial complex I of the ETC and NQO2, leading to the production of mtROS. In contrast, PCr, generated through ETC activity, helps maintain intracellular ATP levels. ROS generated via FADDosome induction, along with ox-mtDNA produced through the TLR4–CMPK2 signaling axis, translocate to the cytosol through the mPTP and VDAC, where they trigger NLRP3 inflammasome activation. Furthermore, cardiolipin interacts with NLRP3 to promote inflammasome assembly. The recruitment of pro-IL-1α to mitochondrial cardiolipin impairs mitophagy and further enhances NLRP3 inflammasome activation during LPS stimulation. **C** Perturbation of lysosomal homeostasis. Various stimuli—including imatinib, masitinib, LL-37, LLME, LPC, *Candida albicans*, H-ferritin, nicotine, particulate matter and crystals, carbon nanotubes, and lecithinase from *Clostridium perfringens*—induce lysosomal damage. This damage leads to the release of cathepsins into the cytosol, which subsequently triggers K⁺ efflux and activates the NLRP3 inflammasome. ATP adenosine triphosphate, Casp8 caspase-8, CLIC chloride intracellular channels, CMPK2 cytidine monophosphate kinase 2, ER endoplasmic reticulum, ETC electron transport chain, FADD fas-associated death domain, GSDMD gasdermin D, GSDME gasdermin E, HBV hepatitis B virus, H-ferritin, heavy chain-ferritin, IP3R2 inositol 1,4,5-trisphosphate receptor type 2, KCNN4 potassium–calcium-activated channel subfamily N member 4, LLME Leu-Leu-O-methyl ester, LPC lysophosphatidylcholine, LPS lipopolysaccharide, mPTP mitochondrial permeability transition pore, mtDNA mitochondrial DNA, N- N terminal, NQO2 quinone oxidoreductase 2, ox-mtDNA oxidized-mitochondrial DNA, PCr phosphocreatine, ROS reactive oxygen species, RTK receptor tyrosine kinase, SFTSV severe fever with thrombocytopenia syndrome virus, TLR4 toll-like receptor 4, TRPM2 transient receptor potential melastatin 2, VDAC voltage-dependent anion channel, VRAC volume-regulated anion channel

Studies have identified stimuli that activate pathways leading to the assembly of the NLRP3 inflammasome, resulting in the release of mature IL-1 and IL-18 and pyroptotic cell death. How diverse stimuli trigger the assembly of the NLRP3 inflammasome complex after priming is unclear. These pathways include 1) perturbation of intracellular ion homeostasis, such as K^+^ efflux, intracellular Ca^2+^ flux, and Cl^−^ efflux; 2) mitochondrial perturbation; and 3) lysosomal destabilization. These second signaling mechanisms have been extensively discussed in review articles by others [14, 57] and us [6]. Therefore, we have focused on recent studies on the major mechanisms of signal 2 activation by updating the latest research (Fig. 2).

### Perturbation of intracellular ion homeostasis

#### K^+^ efflux and the influx of Ca^2+^ and Na^+^

The reduction in intracellular K^+^ appears to be a common mechanism to activate the NLRP3 inflammasome through the convergence of signals from several known NLRP3 agonists, including nigericin, bacterial pore-forming toxins, ATP gating of the P2X7 receptor, and particulate matter [23, 58–60]. These NLRP3-activating stimuli directly disrupt the permeability of the plasma membrane to K^+^, thereby decreasing the cytosolic K^+^ concentration [61]. The ATP-activated P2X7 receptor facilitates K^+^ efflux by opening a nonselective cation channel and a large pore mediated by pannexin-1, which are critical for the activation of caspase-1 mediated by NLRP3 in response to ATP and bacterial components [62, 63]. Furthermore, maitotoxin, a pore-forming toxin, and nigericin, an ionophore, reduce the cellular K^+^ concentration in LPS-stimulated macrophages [59]. The phagocytosis of particulate matter, including monosodium urate, silica, and aluminum hydroxide, and small-molecule lysosomotropic agents induces K^+^ efflux, ultimately leading to the activation of the NLRP3 inflammasome due to lysosomal destabilization [58]. Recently, *Mycobacterium tuberculosis* infection resulted in plasma membrane damage and K^+^ efflux, leading to caspase-1/NLRP3/GSDMD-mediated pyroptosis [64]. Ornithine lipids, which function in the pathogenesis of bacterial infection, trigger TLR4 signaling and K^+^ efflux-dependent NLRP3 inflammasome activation in macrophages [65]. Indeed, NEK7 appears to be a critical K^+^ sensor because NEK7 can be associated with NLRP3 under K^+^ efflux signals to drive inflammation [66]. In a more recent study, K^+^ efflux triggered by P2X7 receptor-mediated signaling led to the NEK7–NLRP3 interaction, which promoted GSDMD-mediated pyroptosis of prostate epithelial cells [67]. Although some explanations have been discussed in the spatiotemporal relationship section, how K^+^ efflux activates the assembly of the NLRP3 inflammasome complex is unclear. However, a recent study showed that low intracellular K^+^ drives the conformational change of ASC oligomers independent of NLRP3, thereby recruiting procaspase-1^CARD^ domain [68]. Because ASC specks are required for other inflammasome scaffolds, including AIM2, NLRC4, or pyrin [68], a more generalized function of K^+^ efflux should be elucidated in other inflammasome complexes. Additionally, there are a few exceptions in which several agonists for NLRP3 inflammasome activation do not depend on K^+^ efflux. For example, short-chain fatty acids, such as butyrate and propionate, induce caspase-8-dependent activation of the NLRP3 inflammasome but are independent of K^+^ efflux [69]. A septin modifier, forchlorfenuron, was found to be an NLRP3 inflammasome activator through a K^+^ efflux-independent mechanism but induced the rearrangement of cytoskeletal proteins to impact mitochondrial function [70]. It is unknown which signals adopt K^+^ efflux, whereas other signals do not; more studies are warranted to differentiate K^+^ dependency in a cell- or stimuli-context manner.

Recent studies have identified K^+^ channels that are critical for K^+^ efflux, e.g., pannexin-1 hemichannels [71] and TWIK2 channels [72]. In alveolar macrophages, extracellular histone-mediated TWIK2 upregulation triggers K^+^ efflux and activates the NLRP3 inflammasome, thereby resulting in excessive lung inflammation during sepsis [73]. Another study characterized the roles of other K^+^ channels, including TREK-1, in activating the NLRP3 inflammasome in alveolar macrophages [74]. Notably, potassium calcium-activated channel subfamily N member 4, a Ca^2+^-activated K^+^ channel, plays a critical role in PIEZO signaling, which converts mechanical inputs into NLRP3-dependent inflammatory pathologies. Mechanistically, potassium calcium-activated channel subfamily N member 4 was found to activate the NLRP3 inflammasome through a Ca^2+^ influx-mediated K^+^ efflux-dependent mechanism [75]. During E-selectin-mediated NLRP3 inflammasome activation, the voltage-gated potassium channel K_V_1.3 promotes ASC oligomerization and GSDMD activation and the release of S100A8/S100A9 [76]. Moreover, DSS, which is used in mouse models of inflammatory bowel disease, augments NLRP3 inflammasome activation through the K^+^ channel KCa3.1 [77]. These data suggest that different stimuli involving K^+^ efflux mechanisms utilize diverse K^+^ channels in a context-dependent manner.

K^+^ efflux is often linked to the influx of Ca^2+^ or Na^+^ signals to activate the NLRP3 inflammasome and maintain cellular ion homeostasis. During this process, intracellular Ca^2+^ flux through calcium-sensing receptor signaling works together with K^+^ efflux [72, 78]. In most cases, Ca^2+^ flux is linked to oxidative stress during inflammation and NLRP3 activation, which involves transient receptor potential melastatin 2 channels [79, 80]. During complement activation, the sublytic membrane attack complex triggers NLRP3 inflammasome activation via Ca^2+^ release from the endoplasmic reticulum (ER) [81]. In addition, the Ca^2+^ channel inositol 1,4,5-trisphosphate receptor type 2 is required for NLRP3 inflammasome activation and GSDMD-triggered pyroptosis in the context of sepsis-induced cardiomyopathy [82]. In the case of microcrystalline stimuli, such as monosodium urate and calcium pyrophosphate (MSU and CPP), the activation of osmosensitive LRRC8 anion channels is critical for NLRP3 inflammasome activation [83]. Notably, intracellular Ca^2+^ flux is required for LRRC8-mediated inflammasome activation in the context of crystal-induced inflammatory pathologies [83].

In macrophages, hepatitis B virus infection enhances hepatic inflammation and NLRP3 inflammasome activation through both K^+^ efflux and Na^+^ influx [84]. A deep understanding of how different signals adopt different channels to utilize several ion fluxes at the same time provides new knowledge of the immune pathogenesis of infectious diseases involving the NLRP3 inflammasome and the development of therapeutics.

#### Cl^−^ efflux

Studies have shown that the suppression of extracellular Cl^−^ levels, in cooperation with other signals, promotes NLRP3 activation and IL-1β secretion. Cl^−^ efflux through chloride intracellular channels functions as a downstream signal of the K^+^ efflux–mitochondrial reactive oxygen species (mtROS) axis in response to NLRP3 agonists, contributing to IL-1β synthesis [85]. Upon LPS stimulation, chloride intracellular channels (CLIC1 and CLIC4) are translocated into the nucleus and induce the transcription of IL-1β and the formation of ASC specks [86]. Recently, Cl^−^ efflux through intracellular Cl^−^ channel proteins was found to function as proximal upstream signals for priming by promoting the NEK7–NLRP3 interaction and inflammasome activation [66]. Importantly, incubation of LPS-primed bone marrow-derived macrophages in Cl^−^-free media induced IL-1β secretion, which was lower than that induced by incubation in K^+^-free media. Both K^+^- and Cl^−^-free conditions induce IL-1β maturation more rapidly and significantly than either ion depletion condition alone does [66]. Moreover, the volume-regulated anion channel, a cell membrane channel that controls cell volume through the transport of Cl^−^ and other organic osmolytes, triggers the activation of the NLRP3 inflammasome by modulating itaconate efflux and mitochondrial damage [87].

As a negative signal involving the Cl^−^ pathway, no lysine kinase 1 (WNK1) was found to be a negative regulator of the NLRP3 inflammasome through the balanced regulation of Cl^−^ efflux and K^+^ efflux [88]. A recent study revealed that the WNK–SPAK–NKCC1 (Na–K–Cl cotransporter) signaling pathway is critical for the anti-inflammatory response in the context of efferocytosis through a Cl^−^-sensing mechanism [89]. A meta-analysis suggested that patients who are administered diuretics that inhibit NKCC2 increase the level of uric acid in the blood, which is potentially linked to the risk of gout, an NLRP3-associated disease [90]. These data strongly suggest that the WNK–NKCC pathway may be associated with the transcriptional regulation of the K^+^ and Cl^−^ transport system in the context of the inflammasome pathway. However, studies must clarify the mechanistic interaction of the WNK pathway in terms of NLRP3 inflammasome regulation. Collectively, these findings suggest that the delicate balance in intracellular ion concentrations is critical for the regulation of NLRP3 inflammasome activation. A detailed understanding of the molecular networks governing ion homeostasis for NLRP3 inflammasome activation will lead to the development of therapeutic approaches for inflammatory and degenerative diseases associated with NLRP3 pathologies.

### Perturbation of mitochondrial homeostasis

Although unclear, persistent mitochondrial dysfunction, excessive mtROS generation, and cytosolic mitochondrial DNA (mtDNA) translocation, potentially linked to the K^+^ and Cl^−^ efflux pathways, can activate the NLRP3 inflammasome [91–95]. Several specific NLRP3 agonists, very high concentrations of chemicals, or pathological conditions are related to mechanisms involving mitochondrial dysfunction in terms of inflammasome activation. The depletion of autophagy proteins, such as microtubule-associated protein 1A/1B light chain 3B, results in the accumulation of dysfunctional mitochondria and cytosolic mtDNA translocation, thereby exaggerating NLRP3 inflammasome responses [96]. Another study revealed that the mitochondrial-specific phospholipid cardiolipin induces ROS-independent NLRP3 inflammasome activation via direct interaction with NLRP3 [97]. Notably, pro-IL-1α translocates to mitochondria upon LPS priming, interacts with cardiolipin, interrupts mitophagy, and enhances NLRP3 activation [98]. However, how cardiolipin drives NLRP3 inflammasome activation is unclear. Recently, the plant growth regulator forchlorfenuron was identified as an NLRP3 inflammasome activator. Like imiquimod, forchlorfenuron impairs the mitochondrial membrane potential and respiration, triggering NLRP3 inflammasome signaling independent of K^+^ efflux, likely through the induction of mitochondrial damage [70]. Aristolochic acid I, the most prevalent aristolochic acid, upregulates the expression of NF-κB and TNF and induces mitochondrial dysfunction, thus activating the NLRP3 inflammasome and leading to ovarian inflammation and fibrosis [99]. However, these conditions regarding NLRP3-associated inflammasome activation should be investigated in the context of other NLRP3-stimulated stimuli to determine whether the responses could be more generalized.

The function of mtROS involvement is controversial. In response to general NLRP3 activators, such as bacterial pore-forming toxins, nigericin, ATP, and particulate matter, mtROS production is dispensable for NLRP3 inflammasome activation [58]. Additionally, ROS were found to be required for the priming, but not the assembly, step of the NLRP3 inflammasome pathway [100]. In a recent study, mitochondrial electron transport chain complexes I, II, III, and V were shown to be required for the sustained activation of the NLRP3 inflammasome through a phosphocreatine dependent, but not ROS-independent, mechanism [101]. However, another study showed that imiquimod and the related molecule CL097 suppress the quinone oxidoreductase NQO2 and mitochondrial complex I, thereby activating the NLRP3 inflammasome through K^+^ efflux-independent and ROS-dependent mechanisms [102]. Notably, the FADDosome, formed by FADD-recruited caspase-8 and RIPK1, induces NLRP3 inflammasome activation through mitochondrial dysfunction and increased mtROS. This results in epithelial cell pyroptosis and mucosal injury in portal hypertensive gastropathy [103]. Future studies must clarify whether mitochondrial dysfunction and mtROS overproduction are central to various pathological models of NLRP3 inflammasome activation.

Recent studies have revealed the mechanistic regulation of cytosolic mtDNA in the activation of the NLRP3 inflammasome and inflammation. MtDNA is easily oxidized, and during apoptosis, cytosolic oxidized mtDNA (ox-mtDNA) can activate the NLRP3 inflammasome through the interaction of ox-mtDNA with the NLRP3 inflammasome [104]. Infections, such as severe fever with thrombocytopenia syndrome virus, trigger mitochondrial dysfunction and ox-mtDNA release, activating the NLRP3 inflammasome [105]. In addition, TLR signals induce ox-mtDNA fragments, activating the NLRP3 inflammasome and causing systemic inflammation [106]. Mechanistically, mitochondrial transcription factor A, the key transcription factor for mtDNA replication and transcription [107], is critical for the production of mtROS and ox-mtDNA. This newly synthesized mtDNA is critical for NLRP3 inflammasome activation [106]. A recent study revealed that cytosolic ox-mtDNA fragments (500–600 bp) translocated through mitochondrial permeability transition pore- and voltage-dependent anion channel (VDAC)-dependent channels can trigger cyclic GMP-AMP synthase-stimulator of interferon genes (STING) pathway signaling and activate the NLRP3 inflammasome [108].

Extracellular mtDNA, which is released into the circulation or enclosed in extracellular vesicles, particularly during mitochondrial damage, activates the NLRP3 inflammasome pathway [109]. GSDMD- and GSDME-mediated pore formation during pyroptosis and apoptosis involves mitochondrial outer membrane permeabilization and disruption of the inner mitochondrial membrane, thereby releasing mtDNA from cells through plasma membrane rupture [110]. In THP-1 macrophages, extracellular mtDNA triggers the priming step of NLRP3 inflammasome activation through TLR9 signaling through increased expression of NLRP3, ASC, and caspase-1, thereby releasing proinflammatory cytokines, including IL-1β, IL-18, and TNF [111]. These findings strongly suggest that cytosolic mtDNA acts as a danger signal to activate the NLRP3 inflammasome, thus promoting pyroptosis and mitochondrial membrane permeabilization, followed by the disruption of mitochondrial and plasma membranes and the release of extracellular mtDNA, ultimately forming a positive feedforward loop. Future studies must answer several unanswered questions. For example, the contribution of mitochondrial destabilization to the second signal of NLRP3 inflammasome activation or the integrated molecular mechanisms of mitochondrial dysfunction and other pathways for inflammasome assembly is unknown.

### Perturbation of lysosomal homeostasis

Frustrated phagocytosis followed by lysosomal damage and cathepsin release into the cytosol is crucial for NLRP3 inflammasome activation. Studies have shown that several damage-associated molecules, including particulate matter, such as uric acid, cholesterol crystals, alum, silica, and asbestos, trigger lysosomal dysfunction [112]. Additionally, carbon-based nanomaterials, such as carbon nanotubes and pristine graphene platelets, activate the NLRP3 inflammasome through lysosomal destabilization and cathepsin B release [113]. Nicotine-induced NLRP3 inflammasome activation depends on lysosomal membrane permeability and is prevented by the lysosome-stabilizing agent dexamethasone [114]. The interaction between cathepsin B and NLRP3 is required for NLRP3 inflammasome assembly and activation upon stimulation by various activators [115]. Moreover, lysosomal damage induced by Leu-Leu-O-methyl ester, a soluble lysosomotropic agent, and lysophosphatidylcholine, a major lipid component of the plasma membrane, requires both signals for full NLRP3 activation, namely, increased K^+^ efflux and lysosomal damage [116, 117]. Nicotine, another damage-associated molecule, activates the NLRP3 inflammasome and IL-1β secretion in aortic vascular smooth muscle cells through lysosomal dysfunction [118]. A recent study revealed that the heavy chain of ferritin activates the NLRP3 inflammasome in hepatic stellate cells through lysosomal damage [119], indicating that hepatic fibrogenesis contributes to chronic liver inflammation through NLRP3 inflammasome activation.

Tyrosine kinase inhibitors, such as imatinib and masitinib, cause lysosomal damage, leading to myeloid cell lysis, K^+^ efflux, and NLRP3 activation [120]. Additionally, the antimicrobial peptide LL-37 promotes NLRP3 inflammasome activation in LPS-primed macrophages through lysosomal destabilization, suggesting a role in the pathogenesis of rosacea [121]. Several bacterial virulence factors activate the NLRP3 inflammasome complex through lysosomal membrane destabilization. For example, *Clostridium perfringens* lecithinase triggers inflammasome activation and GSDMD-independent cell death through lysosomal membrane destabilization [122]. During *Candida albicans* infection, phagosomal rupture and lysosomal damage are critical for NLRP3 activation [123], necessitating further study of these molecular pathways.

More studies are needed to understand how distinct signals provoking lysosomal damage activate the NLRP3 inflammasome and whether other mechanisms, such as ion flux or mtDNA translocation, contribute to inflammasome activation through lysosome destabilization in response to pathogenic and dangerous stimuli.

## Licensing (PTM) regulation of NLRP3 inflammasome activation

**Fig. 3:**
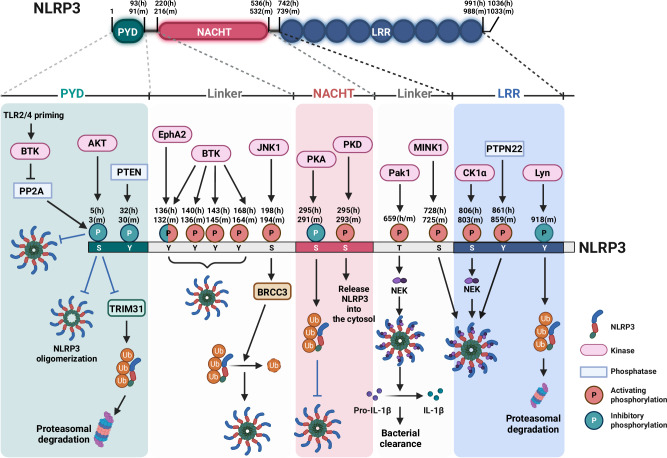
Schematic diagram depicting the phosphorylation or dephosphorylation of the NLRP3 inflammasome. NLRP3 consists of three domains, including the PYD, NACHT, and LRR domains. AKT phosphorylates S5(h)/3(m), hindering TRIM31-associated ubiquitination and degradation as well as NLRP3 oligomerization. PP2A dephosphorylates S5(h)/3(m), facilitating NLRP3 assembly. PTEN dephosphorylates Y32(h)/30(m), triggering NLRP3 inflammasome activation. BTK phosphorylates Y136(h)/132(m), Y140(h)/136(m), Y143(h)/145(m), and Y168(h)/ 164(m), subsequently activating the NLRP3 inflammasome. BTK also suppresses the activation of PP2A, resulting in the inhibition of S5(h)/3(m) dephosphorylation under TLR2/4 priming conditions. EphA2 phosphorylates Y136(h)/132(m), thereby inhibiting inflammasome activation. JNK1 phosphorylates S198(h)/194(m), facilitating the deubiquitination of NLRP3 by BRCC3. The phosphorylation of S295(h)/291(m) by PKA inhibits the NLRP3 inflammasome through ubiquitination. PKD promotes S295(h)/293(m) phosphorylation, releasing NLRP3 for ASC assembly. Pak1 phosphorylates T659 (h/m), leading to the NLRP3–NEK7 interaction, IL-1β maturation, and bacterial clearance. The phosphorylation of NLRP3 at S728(h)/725(m) by MINK1 is required for the priming and activation of the NLRP3 inflammasome. CK1α phosphorylates S806(h)/803(m) and then recruits NEK7 to NLRP3, thus activating the NLRP3 inflammasome. The dephosphorylation of NLRP3 at Y861(h)/859(m) by PTPN22 suppresses inflammasome activation. Lyn kinase phosphorylates Y918(m), resulting in the ubiquitination and subsequent degradation of NLRP3. ASC apoptosis-associated speck-like protein containing a caspase-recruitment domain, BRCC3 BRCA1/BRCA2-containing complex subunit 3, BTK Bruton’s tyrosine kinase, CK1α casein kinase 1 alpha, EphA2 ephrin type-A receptor 2, h human, JNK1 c-Jun N-terminal kinase 1, LRR leucine-rich repeat domain, m mouse, MINK1 misshapen/Nck-interacting kinase-related kinase 1, NEK7 NIMA-related kinase 7, NLRP3 NACHT- leucine-rich-repeat- and pyrin domain-containing protein 3, P phosphorylation, Pak1 p21-activated kinase 1, PKA protein kinase A, PKD protein kinase D, PP2A protein phosphatase 2A, PTEN phosphatase and tensin homolog, PTPN22 protein tyrosine phosphatase nonreceptor 22, PYD pyrin domain, TRIM31 tripartite motif-containing protein 31, TLR toll-like receptor, Ub ubiquitination

**Fig. 4:**
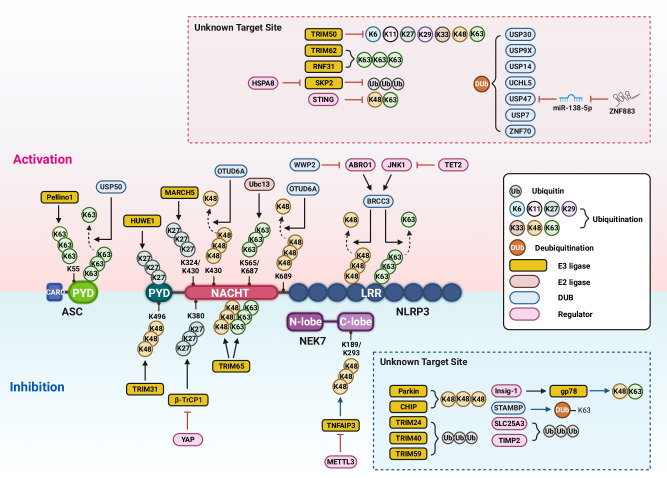
Posttranslational modification of NLRP3 inflammasome components via ubiquitination and deubiquitination. Ubiquitination is a reversible PTM in which Ub is covalently attached to lysine (K) residues of target proteins, particularly involving K48-, K63-, and K27/K29-linked ubiquitination. Pellino-1 promotes NLRP3 inflammasome activation by attaching K63-linked ubiquitin chains to K55 of ASC, whereas USP50 counteracts this process by removing K63-linked chains from ASC, thereby inhibiting activation. HUWE1 and MARCH5 catalyze K27-linked polyubiquitination of NLRP3 at unknown sites within the PYD domain and at K324 and K430 in the NACHT domain, respectively. Ubc13 facilitates K63-linked polyubiquitination of NLRP3 at K565 and K687, while OTUD6A removes K48-linked ubiquitin chains from K430 and K689. Additionally, ABRO1 enhances NLRP3 inflammasome activation by competing with WWP2 for binding to BRCC3. TET2 deficiency promotes JNK1 activation and BRCC3-mediated deubiquitination, further contributing to NLRP3 activation. TRIM50 inhibits NLRP3 ubiquitination, thereby inducing inflammasome activation. Similarly, TRIM62 and RNF31 enhance the K63-linked ubiquitination of NLRP3, promoting its activation. HSPA8 facilitates the degradation of SKP2, which in turn decreases NLRP3 ubiquitination and leads to inflammasome activation. Conversely, STING negatively regulates NLRP3 activation by reducing both K48- and K63-linked ubiquitination of NLRP3. Several deubiquitinating enzymes (DUBs), including USP30, USP9X, USP14, UCHL5, USP47, USP7, and ZNF70, promote NLRP3 inflammasome activation by removing ubiquitin chains from NLRP3. Specifically, USP47 also enhances activation by suppressing miR-138-5p through a ZNF883-dependent mechanism. In contrast, TRIM31 mediates K48-linked polyubiquitination of NLRP3 at K496, targeting it for proteasomal degradation. YAP facilitates NLRP3 degradation via β-TRCP1-mediated K27-linked ubiquitination at K380. TRIM65 induces both K48- and K63-linked ubiquitination of NLRP3, disrupting the NEK7–NLRP3 interaction. TNFAIP3 promotes K48-linked ubiquitination of NEK7 at K189 and K293, leading to its proteasomal degradation, while METTL3 enhances the degradation of TNFAIP3 transcripts. Parkin and CHIP mediate K48-linked polyubiquitination of NLRP3, resulting in its proteasomal degradation in microglia and other contexts, respectively. Furthermore, TRIM24, TRIM40, and TRIM59 promote NLRP3 ubiquitination. The E3 ligase gp78/Insig-1 mediates K48-/K63-linked ubiquitination, thereby inhibiting NLRP3 oligomerization. STAMBP restrains inflammasome activity by removing K63-linked polyubiquitin chains from NLRP3. SLC25A3 promotes NLRP3 ubiquitination, disrupting the NLRP3–NEK7 interaction. Finally, TIMP2 enhances NLRP3 ubiquitination and facilitates its degradation via the autophagy-lysosome pathway. ABRO1 Abraxas brother protein 1, ASC apoptosis-associated speck-like protein containing a CARD, BRCC3 BRCA1/BRCA2-containing complex subunit 3, CARD caspase recruitment domain, β-TrCP1 β-transducin repeat-containing E3 ubiquitin protein ligase 1, Drp1 dynamin-related protein 1, DUB, deubiquitinating enzyme; HSPA8 heat shock protein (HSP) family A member 8, HUWE1 HECT, UBA, and WWE domain-containing E3 ubiquitin protein ligase 1, Insig1 insulin induced gene 1, JNK1 c-Jun N-terminal kinase 1, LRR leucine-rich repeat, MARCH5 membrane-associated ring-CH-type finger 5, METTL3 methyltransferase-like 3, NACHT central NAIP, CIITA, HET-E, and TP1, NEK7 NIMA-related kinase 7,NLRP3 NOD-like receptor family, pyrin domain containing 3, OTUD6A OTU deubiquitinase 6A, PGAM5 phosphoglycerate mutase 5, PYD pyrin domain, RNF31 RING finger protein 31, SERTAD1 SERTA domain-containing 1, SKP2 S-phase kinase-associated protein 2, SLC25A3 solute carrier family 25 member 3, STAMBP STAM-binding protein, STING stimulator of interferon genes, TET2, Tet methylcytosine dioxygenase 2, TNFAIP3 tumor necrosis factor, alpha-induced protein 3 TRIM tripartite motif-containing, Ubc13 ubiquitin-conjugating enzyme E2 13, UCHL5 ubiquitin c-terminal hydrolase L5, USP ubiquitin-specific peptidase, WWP WW domain-containing E3, ZNF70 zinc finger protein 70

**Fig. 5:**
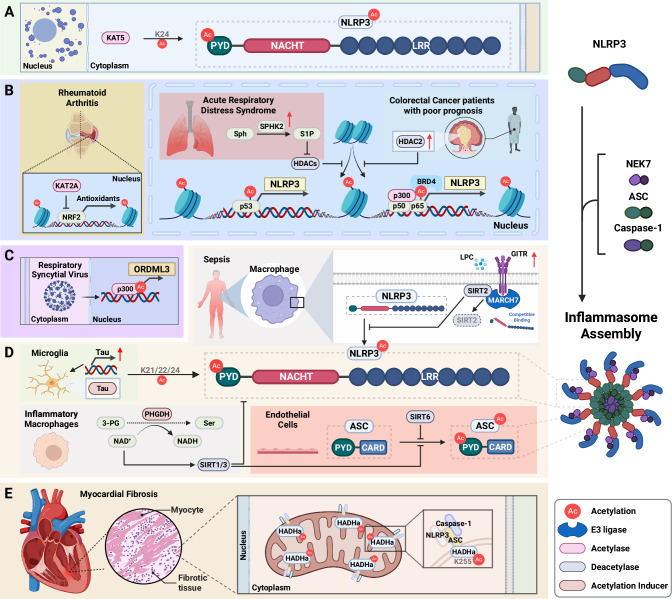
Activation of NLRP3 inflammasome complex through acetylation or deacetylation. Posttranslational modifications via acetylation and deacetylation critically regulate NLRP3 inflammasome activity across various pathological conditions. **A** The acetyltransferase KAT5 directly acetylates NLRP3 at K24. **B** Rheumatoid arthritis, acute respiratory distress syndrome, and colorectal cancer. In rheumatoid arthritis, KAT2A promotes NLRP3 inflammasome activation by suppressing NRF2 transcriptional activity and downregulating antioxidant signaling. In acute respiratory distress syndrome, increased SPHK2 enhances S1P production, which in turn increases p53 acetylation, contributing to inflammasome activation. In colorectal cancer patients with poor prognosis, the upregulated deacetylase HDAC2 represses H3K27 acetylation, thereby modulating NLRP3 transcription through the BRD4/p-p65 complex. **C** Viral infections. During respiratory syncytial virus infection, histone hyperacetylation drives the upregulation of ORMDL3 expression, which in turn regulates NLRP3 expression levels. **D** Microglia in neurodegeneration, inflammatory cells, and endothelial cells. In microglia with elevated tau levels, tau protein acetylates the PYD domain of NLRP3 at K21, K22, and K24 residues. Additionally, PHGDH-mediated serine biosynthesis regulates the acetylation status of both NLRP3 and ASC via NAD+-dependent modulation of SIRT1 and SIRT3 in inflammatory macrophages. In septic macrophages, upregulated GITR competitively binds to MARCH7 instead of NLRP3, leading to the degradation of the deacetylase SIRT2 and resulting in increased NLRP3 acetylation. In endothelial cells, SIRT6 suppresses ASC acetylation, thereby inhibiting inflammasome assembly. **E** Myocardial fibrosis. In myocardial fibrotic cells, acetylation of HADHa at K255 promotes NLRP3 inflammasome activation. ASC adaptor apoptosis-associated speck-like protein containing a caspase-recruitment domain, BRD4 Bromodomain-containing protein 4, GITR glucocorticoid-induced TNFR-related, HADHa hydroxyacyl-CoA dehydrogenase, HDACs histone deacetylases, HDAC2 histone deacetylase 2, KAT2A lysine acetyltransferase 2A, KAT5 lysine acetyltransferase 5, MARCH7 membrane-associated ring-CH-type finger 7, NRF2 nuclear factor erythroid 2-related factor 2, ORMDL3 orosomucoid-like protein 3, PYD pyrin domain, S1P sphingosine-1-phosphate, SIRT2 sirtuin 2, SIRT6 sirtuin 6, SPHK2 sphingosine kinase 2

**Fig. 6:**
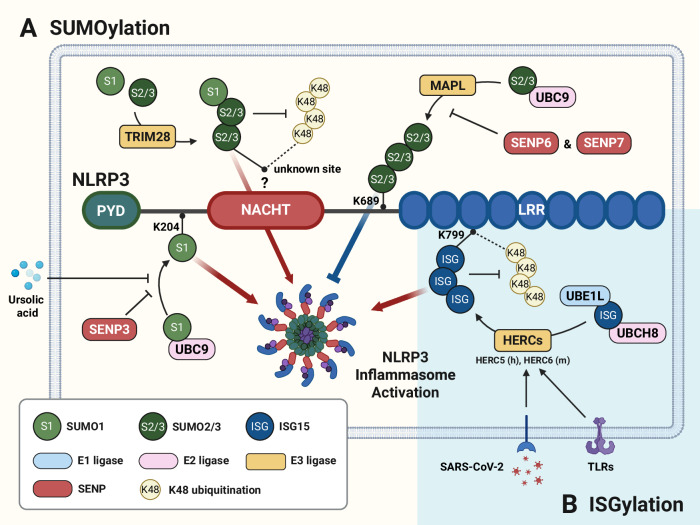
Posttranslational modification of NLRP3 inflammasome components by SUMOylation and ISGylation. **A** The E3 ligase MAPL mediates SUMOylation of NLRP3 at K689 in a UBC9-dependent manner, generating a polymeric SUMO2/3 chain rather than a single SUMO1 moiety, thereby impairing NLRP3 activation. However, the deSUMOylases SENP6 and SENP7 counteract this modification, enhancing NLRP3 inflammasome activation. Additionally, NLRP3 interacts with UBC9, which promotes SUMO1-mediated SUMOylation of NLRP3 at residue K204, resulting in inflammasome activation, whereas SENP3 deSUMOylates NLRP3 to inhibit its activity. Ursolic acid inhibits SUMO1-mediated SUMOylation of NLRP3, thereby preventing its activation and limiting excessive inflammation. Furthermore, TRIM28 interacts with NLRP3 to facilitate SUMOylation at an as-yet unidentified site, which inhibits K48-linked ubiquitination and subsequent proteasomal degradation of NLRP3. **B** ISGylation is another PTM in which ISG15 is conjugated to target proteins through an enzymatic cascade involving E1 (UBE1L), E2 (UBCH8) and E3 (HERCs) ligases. TLR activation and SARS-CoV-2 infection upregulate HERC expression (HERC5 in humans and HERC6 in mice), allowing ISG15 to conjugate to NLRP3 at K799. This inhibits K48-linked ubiquitination, which leads to increased NLRP3 inflammasome activation. HERC HECT domain- and RCC1-like domain-containing proteins, IGF15 interferon-stimulated gene 15, LRR leucine-rich repeat, MAPL mitochondrial-anchored protein ligase, NACHT central NAIP, CIITA, HET-E, and TP1, PYD pyrin domain, SENP Sentrin/SUMO-specific protease, SUMO small ubiquitin-like modifier, TLRs toll-like receptors, TRIM28 tripartite motif-containing protein 28, UBC9 ubiquitin-conjugating enzyme E2 9

**Fig. 7:**
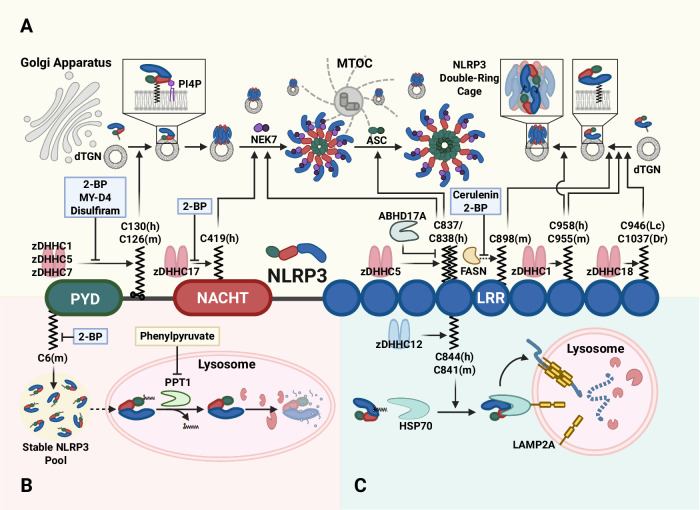
Palmitoylation of NLRP3 regulates inflammasome activation. **A** Palmitoylation at C130(h)/C126(m), C898(m), C958(h)/C955(m), and the teleost-specific sites C946(Lc NLRP3)/C1037(Dr NLRP3) facilitates NLRP3 trafficking to PI4P-enriched, dTGN membranes, promoting inflammasome activation. Among these, palmitoylation at C958(h)/C955(m) stabilizes the double-ring, cage-like oligomer during the priming phase. Palmitoylation at C419(h) and C837/838(h) enhances NEK7 interaction, with C837/838(h) further promoting ASC recruitment. These modifications are catalyzed by the palmitoyltransferases zDHHC1, zDHHC5, zDHHC7, zDHHC17, and zDHHC18. FASN supports palmitoylation at C898(m), a process inhibited by cerulenin, while ABHD17A depalmitoylates C837/838(h). Palmitoylation inhibitors such as 2-BP (a general and non-selective palmitoylation inhibitor), MY-D4 (a zDHHC-targeting tool compound), and disulfiram (a thiol-reactive compound that blocks palmitoylation) suppress NLRP3 inflammasome activation. **B** Additionally, palmitoylation at C6(m)/C8(h) protects NLRP3 from lysosome-mediated autophagic degradation, stabilizing the intracellular NLRP3 protein pool and indirectly promoting inflammasome readiness. In diabetic wound healing, the depalmitoylase PPT1 antagonizes this stabilization, whereas phenylpyruvate inhibits PPT1 activity, thereby enhancing NLRP3 persistence. **C** Conversely, palmitoylation at C844(h)/C841(m), mediated by zDHHC12, facilitates HSC70 binding and directs NLRP3 to LAMP2A-dependent chaperone-mediated autophagy, thereby restraining excessive inflammasome activation. 2-BP 2-bromopalmitate, ABHD17A alpha/beta hydrolase domain containing 17A, Dr *Danio rerio*, dTGN dispersed trans-Golgi network, FASN fatty acid synthase, HSP70 heat shock protein 70, LAMP2A lysosome-associated membrane protein 2A, Lc, *Larimichthys crocea*, MTOC microtubule-organizing center, PI4P phosphatidylinositol-4-phosphate, PPT1 palmitoyl-protein thioesterase 1, zDHHC zinc finger DHHC (Asp-His-His-Cys motif-containing) domain-containing palmitoyltransferase

Growing evidence indicates the role of PTMs in the components of the NLRP3 inflammasome, which are essential for its activation checkpoint, also known as licensing [21, 22, 124–126]. Specifically, PTMs, such as phosphorylation or dephosphorylation (Fig. 3), ubiquitination or deubiquitination (Fig. 4), acetylation or deacetylation (Fig. 5), SUMOylation (Fig. 6), citrullination, ISGylation (Fig. 6), and palmitoylation (Fig. 7), are involved in maintaining the NLRP3 inflammasome in an autosuppressed yet signal-competent state or activating inflammasome assembly [94]. Other lesser-known PTMs, including ADP-ribosylation [127, 128], nitrosylation [56, 129], and prenylation [130, 131], are not discussed in this review. Although each modification appears necessary, coordinating them to determine a unified regulatory model for NLRP3 activation remains challenging and potentially varies across cell types, stimulants, and contexts.

### Phosphorylation or dephosphorylation of the NLRP3 inflammasome

Numerous sites and enzymes regulate NLRP3 phosphorylation, which is vital for the licensing step. Given the extensive work on the phosphorylation or dephosphorylation of NLRP3 and other components, we addressed this aspect depending on the structure of NLRP3 (Fig. 3).

At the N-terminal PYD domain, phosphorylation of residue S5 (murine S3) is pivotal for ASC speck formation and IL-1β secretion, whereas the S5D variant hinders NLRP3 self-assembly [132]. S5 phosphorylation involves two distinct enzymes that optimally activate the NLRP3 inflammasome. AKT, a serine/threonine-specific protein kinase, phosphorylates S5, hindering tripartite motif-containing protein (TRIM) 31-mediated ubiquitination and degradation, as well as NLRP3 oligomerization [133, 134], whereas protein phosphatase 2A (PP2A) dephosphorylates S5, facilitating NLRP3 assembly [135]. PYD Y32 (murine Y30) is critical for facilitating phosphatase and tensin homolog (PTEN)-mediated dephosphorylation, thereby triggering NLRP3 inflammasome activation and ultimately enhancing antitumor immunity [136].

Bruton’s tyrosine kinase (BTK) phosphorylates NLRP3 at multiple sites, including Y136, Y140, Y143, and Y168 (murine Y132, Y136, Y145, and Y164, respectively), within the linker region connecting the PYD and NACHT domains, thereby resulting in NLRP3 inflammasome activation [137, 138]. Targeting BTK with pharmacologic inhibitors, such as the US Food and Drug Administration-approved inhibitor ibrutinib, is promising for suppressing exaggerated inflammatory responses stemming from NLRP3 inflammasome activation [139]. However, BTK inhibits NLRP3 inflammasome activation, particularly under conditions of TLR4 or TLR2 priming in immune cells during physiological NLRP3 inflammasome activation in the absence of a costimulatory signal [140, 141]. This inhibitory action is mediated by the BTK–NLRP3 interaction, which impedes inflammasome assembly by blocking the PP2A-mediated dephosphorylation of S5 in the PYD of NLRP3 [141]. This inhibitory function of BTK is pivotal for understanding the spontaneous onset of colitis observed in patients with X-linked agammaglobulinemia who harbor *BTK* mutations [141]. Overall, these findings highlight BTK as an emerging and multifunctional kinase involved in the regulation of NLRP3 inflammasome activation through site-specific modulation of NLRP3 function and modification of a polybasic linker, which directs the association of NLRP3 with the Golgi and the nucleation of the inflammasome [138].

In the linker domain structure of PYD/NACHT, Y136 (murine Y132) is phosphorylated by ephrin type-A receptor 2 (EphA2), a transmembrane tyrosine kinase expressed selectively in airway epithelial cells in both humans and mice [30]. Depletion of EphA2 exacerbated lung inflammation in a murine reovirus infection model by activating the NLRP3 inflammasome. EphA2 interacts with NLRP3, phosphorylating Y132 in the PYD/NACHT interdomain structure, thereby regulating inflammasome activation [30].

A recent study revealed that S198 (murine S194), located between the PYD and NACHT domains [142], plays a crucial role in NLRP3 inflammasome activation [143]. In addition, c-Jun N-terminal kinase (JNK) 1 is responsible for S198 phosphorylation, facilitating the deubiquitination of NLRP3 by BRCA1/BRCA2-containing complex subunit 3 (BRCC3), which is a prerequisite for inflammasome assembly. Blocking S194 phosphorylation suppresses NLRP3 inflammasome activation in cryopyrin-associated periodic syndrome (CAPS) patients, suggesting its importance as a therapeutic target for treating NLRP3-related diseases [143]. Recently, JNK1 and JNK2 were found to differentially regulate the biphasic function of JNK, enabling the licensing of NLRP3 inflammasome activation to facilitate inflammasome assembly and GSDMD-mediated cell death, respectively [144].

NLRP3 S295 (murine S291) is phosphorylated by cyclic adenosine monophosphate-dependent protein kinase A in response to bile acids, which inhibits the inflammasome through NLRP3 ubiquitination [145]. Protein kinase D, which is recruited by diacylglycerol at the Golgi, phosphorylates NLRP3 at S295 (murine S293), releasing NLRP3 into the cytosol for ASC assembly [146]. In human and murine macrophages, T659 between the NACHT and LRR domain of NLRP3 was found to be critical for NLRP3 inflammasome activation involving the activity of the Rho GTPase Rac2 [147]. The activity of p21-activated kinase 1 is required for the T659 phosphorylation of NLRP3, leading to the NLRP3–NEK7 interaction and IL-1β maturation. The p21-activated kinase 1–NLRP3 axis contributes to bacterial clearance of the uropathogenic *Escherichia coli* in vivo [147]. The phosphorylation of NLRP3 at S728 (murine S725) by misshapen/Nck-interacting kinase-related kinase 1 is imperative for the priming and activation of the NLRP3 inflammasome. This process relies on the interaction between misshapen/Nck-interacting kinase-related kinase 1 and the linker domain of LRR domain within NLRP3 [148].

The phosphorylation of NLRP3 at S806 (murine S803) within its LRR domain is emerging as a key checkpoint for NLRP3 inflammasome assembly. This NLRP3 S803 phosphorylation event is regulated by casein kinase 1 alpha 1 and is crucial for recruiting NEK7 to NLRP3, thereby activating the NLRP3 inflammasome in macrophages and in vivo [149]. Interestingly, the NLRP3 S803A mutant retains the ability to recruit NEK7 but fails to activate the NLRP3 inflammasome, suggesting that NEK7 recruitment alone is insufficient for inflammasome activation in a context-dependent manner. The subsequent dephosphorylation of S806 is essential for initiating the assembly of the NLRP3 inflammasome to achieve full activation [149].

Within the LRR domain of NLRP3, phosphorylation at Y861 (murine Y859) is critical for inflammasome activation across various cell types, including THP-1 cells, human peripheral blood mononuclear cells, and murine bone marrow-derived dendritic cells [150, 151]. Protein tyrosine phosphatase nonreceptor 22 (PTPN22) has emerged as a key regulator that modulates Y861 phosphorylation during inflammasome activation [150]. This insight highlights the importance of PTPN22 variants associated with chronic inflammatory disorders because they facilitate the dephosphorylation of NLRP3 upon inflammasome induction, thus enabling efficient NLRP3 activation and subsequent IL-1β release [150]. Patients with inflammatory bowel disease, which involves an autoimmunity-associated PTPN22 variant, exhibit increased IL-1β levels, underscoring the role of Y861 phosphorylation in regulating NLRP3 and preventing aberrant inflammasome activation [150]. Residues S894 and S898 (murine S891 and S895, respectively) within the LRR domain of NLRP3 are essential for interaction with the E3 ligase β-transducin repeat-containing E3 ubiquitin (Ub) protein ligase 1 (β-TrCP1) [152]. However, the kinase responsible for these phosphorylation sites was not identified in this study. The phosphorylation of residue Y918 within the NLRP3 LRR domain is required for NLRP3 ubiquitination and subsequent degradation, thus controlling inflammasome activity [153]. Lyn kinase plays a role in this phosphorylation, and its deficiency increases IL-1β production and susceptibility to septic shock [153].

Although the specific phosphorylation sites involved remain elusive, other kinases have been implicated in regulating NLRP3 inflammasome priming and licensing. Notably, inhibitory kappa B kinase (IKK) β facilitates NLRP3 inflammasome priming and assembly by positioning NLRP3 to phosphatidylinositol-4-phosphate, which is enriched in the trans-Golgi network. Inhibiting or silencing IKKβ attenuated NLRP3 inflammasome activation [154, 155]. The stimulation of human monocytes with TLR2 agonists triggered the alternative “one-step” NLRP3 inflammasome activation pathway. In this context, TLR2-dependent signaling pathways through TRAF6–TAK1–IKKβ are essential for activating the NLRP3 inflammasome in human monocytes [156]. The kinases TANK-binding kinase 1 and IKKε play a negative regulatory role in NLRP3 inflammasome activation, counteracting the function of PP2A, which confers an “ON” switch for NLRP3 licensing [135]. However, the phosphorylation sites targeted by these two kinases should be clarified in future studies. These findings collectively suggest that different protein kinases play both positive and negative regulatory roles during the priming and licensing stages of NLRP3 inflammasome activation. These fine-tuning processes are likely critical for regulating the excessive activation of the NLRP3 inflammasome, which can contribute to disease pathology. Further research must determine the mechanisms by which various kinases modulate the NLRP3 inflammasome in different contexts or cell types.

### Ubiquitination or deubiquitination of the NLRP3 inflammasome

Ubiquitination and deubiquitination are crucial protein modifications involved in various biological functions, including the modulation of the composition of the NLRP3 inflammasome [157]. The ubiquitin–proteasome system orchestrates target protein modifications, impacting cellular processes such as localization, activation, function, and degradation [158]. Ubiquitination involves the formation of E1, E2, and E3 ligases, facilitating thioester bond formation, coupling, and the transfer of activated Ub to substrates. Ub has multiple residues and is subject to various types of ubiquitination, such as monoubiquitination, multimeric ubiquitination, and polyubiquitination, which are reversible by deubiquitinating enzymes (DUBs). DUBs, which feature diverse structural domains, finely regulate substrate biological processes [159]. Figure 4 depicts the ligases and DUBs that interact with the ubiquitination and deubiquitination sites of NLRP3, as well as regulators with unidentified targets.

#### Ubiquitination of NLRP3 inflammasome components

Numerous enzymes, such as E3 Ub ligases, which target NLRP3 proteins or their components, have been identified as key regulators of NLRP3 inflammasome degradation. The E3 Ub ligase HUWE1 was found to interact with AIM2, NLRP3, and NLRC4 through the HIN domain of AIM2 and the NACHT domains of NLRP3 and NLRC4, which is mediated by the HUWE1 BH3 domain [160]. This interaction promotes the K27-linked polyubiquitination of AIM2, NLRP3, and NLRC4, which are essential for inflammasome assembly, ASC speck formation, and IL-1β production upon bacterial infection [160]. The mitochondria-associated E3 ligase membrane-associated RING-CH-type finger (MARCH) 5 was shown to promote NLRP3 inflammasome activation by interacting with the NACHT domain of NLRP3 and facilitating K27-linked polyubiquitination of residues K324 and K430 of NLRP3 [161]. This process is crucial for the formation of the NLRP3–NEK7 complex and the subsequent oligomerization of NLRP3 on the mitochondria [161].  The binding of the E3 ligase β-TrCP1 to NLRP3 results in K27 ubiquitination at K380 and the subsequent proteasomal degradation of NLRP3. YAP, a critical component of the Hippo pathway, competes with β-TrCP1 for interaction with NLRP3, thereby promoting the NLRP3 inflammasome and associated disease pathology [152]. Ubc13, an E2 Ub-conjugating enzyme, interacts with NLRP3 and enhances its K63-linked polyubiquitination [162]. Notably, the NLRP3 residues K565 and K687 serve as specific sites for Ubc13-mediated K63-linked polyubiquitination during the activation of the NLRP3 inflammasome [162]. In contrast, parkin, an E3 Ub ligase, plays a crucial role in facilitating the degradation of NLRP3 through K48-linked polyubiquitination [163]. Parkin-knockout mice presented heightened assembly of microglial NLRP3, leading to intensified motor impairment and loss of dopaminergic neurons in vivo. Therefore, strategies aimed at facilitating the parkin-mediated degradation of NLRP3 could provide a therapeutic approach for Parkinson’s disease [163]. The carboxy terminus of the Hsc70-interacting protein, which functions as a U-box E3 ligase, has been reported to interact with and ubiquitinate NLRP3 through K48 ubiquitination but not K63 ubiquitination, leading to its subsequent degradation through the proteasome [164]. This interaction restrains redox-related damage in septic acute kidney injury (AKI) by suppressing NLRP3 inflammasome-mediated pyroptosis [164]. These findings suggest that targeting the Ub-conjugating enzymes involved in the regulation of NLRP3 ubiquitination is a promising therapeutic approach for addressing the pathological consequences of dysregulated NLRP3 inflammasome activation.

Several TRIM family members, notable for their E3 Ub ligase activity, play critical roles in NLRP3 inflammasome activation. TRIM24 facilitates the ubiquitination of NLRP3, thus attenuating NLRP3 inflammasome activation and pyroptosis [165]. Notably, reduced expression of TRIM24 was observed in the ectopic endometria of patients with endometriosis, leading to increased NLRP3 inflammasome-mediated inflammation and pyroptosis [165]. The transcription factor Cdx2 was shown to regulate TRIM31 expression, thereby mitigating intestinal inflammation triggered by NLRP3 inflammasome activation [166]. TRIM31 negatively regulates the activation of the NLRP3 inflammasome in *Helicobacter pylori*-associated gastritis by modulating ROS production and autophagy flux in gastric epithelial cells [167]. TRIM31 enhances K48-linked polyubiquitination and subsequent proteasomal degradation of NLRP3 [168]. This effect was coupled with increased interaction between NLRP3 and TRIM31 in intestinal epithelial cells, thereby dampening NLRP3-mediated inflammatory responses in the intestine [168]. TRIM40 interacts with NLRP3, acting as a negative regulator of NLRP3 inflammasome activation and the proliferation of glomerular mesangial cells in response to IgA1 [169]. In cerebral ischemia‒reperfusion (IR) injury, TRIM59 inhibits NLRP3 inflammasome activation and pyroptotic cell death by interacting with NLRP3 and suppressing it through NLRP3 ubiquitination [170]. TRIM65 negatively regulates NLRP3 inflammasome activation by binding to the NACHT domain of NLRP3, thereby facilitating NLRP3 ubiquitination and impeding the NEK7–NLRP3 interaction [171].

In contrast, several members of the TRIM family have been implicated in augmenting the activating functions of the NLRP3 inflammasome in various contexts. For example, TRIM50 interacts with NLRP3 through its interesting new gene (RING) domain, promoting NLRP3 oligomerization. This interaction leads to the inhibition of NLRP3 ubiquitination and the amplification of inflammasome activation [172]. Furthermore, TRIM21 induced GSDMD ubiquitination in vitro; however, TRIM21 promoted GSDMD N-terminal fragment aggregation during pyroptosis by interacting with GSDMD independently of its RING domain, allowing GSDMD to remain stable in resting cells [173]. In the cerebral IR model, TRIM62 expression was upregulated in response to oxygen and glucose deprivation, playing a crucial role in regulating the activation of the NLRP3 inflammasome through the K63 Ub of TRIM62 and its interaction with NLRP3 [174]. However, the types of TRIM family members involved in regulating NLRP3 ubiquitination under distinct conditions are unclear. Moreover, the TRIM molecules that contribute to the regulatory pathways of NLRP3 inflammasome activation in different scenarios are unknown. Studies have highlighted the importance of TRIM family members in modulating NLRP3-mediated inflammation across diverse cell types and tissues in response to various stimuli.

Pellino-1 is an E3 Ub ligase that plays a role in NLRP3 inflammasome activation but not in the AIM2, NLRP1, or NLRC4 inflammasome [175]. Mechanistically, Pellino-1 connects the K63 Ub chain to K55 in ASC, leading to increased oligomerization and interaction with NLRP3, ultimately activating the inflammasome [175]. Another study revealed that Pellino-1, along with NLRP3, is overexpressed in the renal tissue of septic patients and mice [176]. This elevation corresponds to NLRP3-associated pathologies in renal tissues during AKI, suggesting that Pellino-1 is a promising therapeutic target [176]. In ulcerative colitis, RING finger protein 31, an E3 Ub ligase, interacts with NLRP3 through the RING-between-RING finger domain, resulting in K63-linked ubiquitination of NLRP3 and its subsequent stabilization [177].

The membrane-bound E3 Ub ligase gp78 triggers the ubiquitination of NLRP3, reducing its oligomerization and subcellular translocation [178]. ER membrane protein insulin-induced gene 1 facilitates the gp78–NLRP3 interaction and gp78-mediated NLRP3 ubiquitination, emphasizing gp78’s role in suppressing NLRP3 inflammasome activation by regulating its ubiquitination [178]. A recent study revealed that the interaction between MARCH2 and phosphoglycerate mutase 5 (PGAM5) affects mitochondrial antiviral signaling protein–NLRP3 signaling [179]. This interaction induced the K48-linked polyubiquitination of PGAM5, leading to its proteasomal degradation. As a result, PGAM5–mitochondrial antiviral signaling protein cocondensation decreases, inhibiting NLRP3 inflammasome activation and cardiomyocyte pyroptosis [179]. Further investigations are warranted to elucidate the novel regulatory mechanisms underlying NLRP3 ubiquitination. These findings provide insights for developing therapeutics for NLRP3 inflammasome-associated inflammatory diseases.

#### Deubiquitination of the NLRP3 inflammasome

Several recent studies have highlighted the involvement of DUBs in promoting NLRP3 inflammasome activation, although the specific targeted sites on NLRP3 or the inflammasome components remain unclear. Early studies highlighted the importance of nontranscriptional priming and deubiquitination of NLRP3 in activating the NLRP3 inflammasome [180, 181]. Notably, BRCC3 (also known as BRCC36 in human) is a crucial DUB regulator of NLRP3 activity, operating through the formation of the cytosolic BRCC3-containing BRCC36 isopeptidase complex (BRISC) [181]. Further research demonstrated that priming signals initiate the association of Abraxas brother protein 1, a component of the BRISC, with NLRP3 in an S194 phosphorylation-dependent manner. This interaction facilitates the recruitment of the BRISC to deubiquitinate the K63-linked Ub chains of NLRP3 upon stimulation with NLRP3 activators [182]. A recent study revealed that TET2 deficiency, an epigenetic regulator of DNA methylation, promoted JNK activation and BRCC3-mediated DUB of the NLRP3 inflammasome, accelerating atherosclerosis and the formation of neutrophil extracellular traps [183]. This pathological increase in inflammasome activation due to TET2 deficiency can be counteracted by holomycin, a BRCC3 deubiquitinase inhibitor, highlighting its potential as a therapeutic and preventive agent for cardiovascular diseases associated with inflammasome activation [183].

Studies have shown the roles of various Ub-specific protease (USP) members in the regulation of NLRP3 inflammasome activation. USP30 interacts with and deubiquitinates NLRP3, thereby facilitating the activation of the NLRP3 inflammasome [184]. Considering that USP30 is upregulated in the skin tissues of patients with diabetic foot ulcers, targeting USP30 with the inhibitor MF-094 seems to be promising for promoting wound healing in diabetic rats and suppressing NLRP3 inflammasome activation [184]. USP50 levels are elevated in both a duodenogastric reflux model and cases of human bile reflux gastritis [185]. USP50 was shown to be essential for increasing NLRP3 inflammasome activation and the release of high mobility group box 1, which is related to tumorigenesis. A unique mechanism by which USP50 activates the NLRP3 inflammasome is based on its interaction with and deubiquitination of ASC, although the PTM sites are unclear [185]. Furthermore, USP25 is significantly upregulated in acute pancreatitis models and is implicated in the activation of GSDMD-mediated pyroptotic cell death in acinar cells during acute pancreatitis. USP25 interacts with Krüppel-like factor 4, promoting its stabilization through deubiquitination, thereby suppressing miR-10a-5p, which targets GSDMD [186]. These findings suggest that various DUBs play crucial roles in activating the NLRP3 inflammasome and pyroptosis across cell types and contexts by directly or indirectly modulating the ubiquitination of NLRP3 and its associated proteins.

USP9X has emerged as a key player in intensifying acute lung injury by facilitating NLRP3 inflammasome activation and subsequent pyroptosis in alveolar epithelial cells [187]. Additionally, USP14 enhances the NLRP3 inflammasome and pyroptotic activation in annulus fibrosus cells in the context of intervertebral disc degeneration models [188]. During hepatitis C virus infection, the Ub C-terminal hydrolase L5-mediated deubiquitination of NLRP3 enhances NLRP3 inflammasome activation in hepatocytes, underscoring its pivotal role in the pathogenesis of hepatitis C virus infection-induced liver diseases, including fibrosis, cirrhosis, and cancer [189]. Moreover, elevated levels of USP47 are correlated with NLRP3 inflammasome activation and the progression of epilepsy [190]. Mechanistically, the overexpression of zinc finger protein (ZNF) 883, a long noncoding RNA (lncRNA) that targets miR-138-5p, increased USP47 expression. ZNF883-induced USP47 expression suppressed NLRP3 ubiquitination and promoted epilepsy by sponging miR-138-5p [190]. Notably, other reports have highlighted the involvement of DUBs in pathways beyond the inflammasome complex. Through interaction with the C-terminal ligand-binding domain of the vitamin D receptor (VDR), DUB3 initiates the activation of VDR signaling, which is associated with antioxidant responses [191]. This ability to regulate VDR signaling holds significant promise for mitigating the progression of Parkinson’s disease [191]. Further studies are needed to identify novel regulators of DUBs and their activities, which influence ubiquitination during NLRP3 inflammasome activation.

A recent study on osteosarcoma progression revealed that the activation of mTOR complex 1 signaling by 17β-estradiol increased the stability of USP7 mRNA, which interacts with NLRP3, facilitating its deubiquitination through K48 ubiquitination [192]. In the context of gouty arthritis, increased levels of Drp1 are implicated in the activation of the NLRP3 inflammasome and associated pathologies [193]. USP16 plays a crucial role by interacting with Drp1, leading to its deubiquitination and stabilization, consequently activating the NLRP3 inflammasome [193]. Ovarian tumor deubiquitinase 6A, which functions as a DUB, was found to bind the NACHT domain of the NLRP3 inflammasome [194]. It targets the K48-linked poly-Ub chains on NLRP3, cleaving them at residues K430 and K689, thereby promoting the stability of NLRP3. This action increases IL-1β levels and inflammation, thereby worsening DSS-induced colitis [194]. Recently, ZNF70 was found to interact with NLRP3 to reduce its K48-linked ubiquitination, resulting in the activation of the NLRP3 inflammasome [195]. Considering that ZNF70 expression is elevated in colorectal cancer (CRC), ZNF70 could be a promising target for CRC treatment [195].

Several studies have highlighted the negative regulatory role of DUBs in NLRP3 inflammasome activation. Depletion of the DUB STAM-binding protein results in an exaggerated inflammatory response characterized by increased NLRP3 K63-linked polyubiquitination and increased NLRP3 inflammasome activation [196]. The E3 ubiquitin ligases WW domain-containing protein (WWP) 2 play critical roles in the ubiquitination and subsequent degradation of the DUB BRCC3, suppressing NLRP3 inflammasome activation [197]. Abraxas brother protein 1 promoted NLRP3 inflammasome activation by competing with WWP2 for BRCC3 binding, thereby inhibiting WWP2-mediated ubiquitination of BRCC3 and increasing BRCC3 stability [197]. Importantly, the *N*6-methyladenosine (m^6^A) "reader" protein YTHDF1 increases the translation of the E3 Ub ligase WWP1. This in turn induces the ubiquitination of NLRP3, consequently reducing the NLRP3 inflammasome and pyroptotic activation [198]. Furthermore, USP22 was found to negatively regulate NLRP3 inflammasome activation by facilitating the K27- and K48-linked deubiquitination of autophagy-related 5 at K118, thus promoting its stabilization and activating autophagy [199]. Future research is warranted to identify additional negative DUBs that engage in nondegradative ubiquitination of NLRP3, with the aim of identifying therapeutic targets for excessive inflammasome activation and mitigating injurious IL-1β-induced inflammatory signaling.

#### Regulators of ubiquitination of the NLRP3 inflammasome

In addition to E3 Ub ligases, several positive and negative regulatory molecules modulate the ubiquitination of NLRP3 or other NLRP3 inflammasome complex components. A recent study revealed that the inhibition of heat shock protein (HSP) family A member 8 augmented lung injury by promoting NLRP3 ubiquitination through the degradation of the E3 Ub ligase S-phase kinase-associated protein 2 [200]. Methyltransferase-like (METTL) 14-mediated m^6^A modification promoted NLRP3 inflammasome activation during arsenic-induced hepatic insulin resistance [201]. Moreover, METTL3–METTL14 complex-mediated m^6^A modification reduces the stability of tumor necrosis factor alpha-induced protein 3 (also known as A20), a deubiquitinating enzyme [202]. Silencing METTL3 led to A20 accumulation, which resulted in increased NEK7 ubiquitination and, consequently, impaired NLRP3 inflammasome assembly, indicating that METTL3-mediated m^6^A modifications are essential for NLRP3 inflammasome activation [202]. A study published around the same time revealed that A20 plays an important role in regulating NEK7 ubiquitination. A20 was found to bind directly to NEK7, mediating its K48-linked ubiquitination and thus targeting NEK7 for proteasomal degradation [203].

A recent study revealed that solute carrier family 25 member 3, a phosphate carrier protein located in the mitochondria, serves as a crucial negative regulator of NLRP3 inflammasome activation [204]. Mechanistically, solute carrier family 25 member 3 interacts with NLRP3, disrupting the NLRP3–NEK7 interaction and enhancing the ubiquitination of NLRP3 [204]. Other reports have underscored the presence of negative regulators influencing the activation of NLRP3 inflammasome-associated pyroptosis across pathological contexts. In a model of sepsis-associated AKI, a tissue inhibitor of metalloproteinases 2 plays a crucial role in mitigating pyroptosis and suppressing renal damage [205]. This effect is partially reliant on the tissue inhibitor of metalloproteinases 2-mediated augmentation of NLRP3 ubiquitination, leading to subsequent degradation through the enhancement of intracellular cyclic adenosine monophosphate [205]. These findings highlight the importance of further research into the mechanisms regulating NLRP3 ubiquitination, which influences the attenuating effects of these regulators in a wide range of disease pathologies, including cancer.

Bood POZ-containing gene type 2, which acts as a scaffold for the cullin-3 E3 Ub ligase, interacts with NLRP3, promoting its degradation through cullin-3 recruitment and thus suppressing NLRP3 inflammasome activation [206]. However, SERTA domain-containing protein 1, an adaptor protein, is involved in regulating K63-linked NLRP3 polyubiquitination by cullin-1 through binding to NLRP3 and disrupting its interaction with cullin-1 [207]. A deficiency in SERTA domain-containing protein 1 suppressed NLRP3 inflammasome activation, IL-1β and IL-18 secretion, and GSDMD cleavage in bone marrow-derived macrophages [207]. Further studies are needed to identify molecules that play distinct roles in regulating cullin-1-mediated NLRP3 polyubiquitination, contributing to our understanding of the complex regulatory networks governing NLRP3-mediated inflammasome activation.

STING is involved in the cytosolic DNA-sensing pathway [208, 209]. STING promoted NLRP3 deubiquitination and localization in the ER during HSV-1 infection, activating the NLRP3 inflammasome [210]. STING causes tubular cell inflammation and pyroptosis by activating ER stress in LPS-induced AKI [211]. According to recent research, the STING pathway is associated with Pellino-1 in mice and patients with AKI, suggesting that Pellino-1 may be involved in STING-mediated deubiquitination of NLRP3 in this context [176]. Future studies are warranted to explore the specific role of STING in regulating the ubiquitination of the NLRP3 inflammasome.

### Acetylation or deacetylation of the NLRP3 inflammasome

Lysine acetylation, which is mediated by lysine acetyltransferases (KATs) and reversed by lysine deacetylases, plays a critical role in inflammasome regulation (Fig. 5). Recent studies suggest that the full activation of the NLRP3 inflammasome depends on the acetylation of NLRP3 and other components of the inflammasome complex [212–214]. The acetylation of NLRP3 at specific lysine residues (K21, K22, and K24 in mice; K23, K24, and K26 in humans) promotes its activation, which is controlled by sirtuin (SIRT) 2 [215]. This acetylation is required for the activation of the NLRP3 inflammasome and is linked to aging-related inflammation and insulin resistance [215]. KAT5-mediated acetylation of NLRP3 at K24 induces its oligomerization and subsequent assembly of the NLRP3 inflammasome [212]. Tau acetylates K21, K22, and K24 within the NLRP3 PYD domain, activating the inflammasome [213]. Human tau overexpression in mouse hippocampal CA1 neurons impairs cognition and triggers tau transmission to microglia, inducing NLRP3 acetylation and inflammasome activation. In mice, the tau-NLRP3-binding peptide disrupts this interaction, inhibiting NLRP3 acetylation and inflammasome activation in microglia and thus ameliorating cognitive decline [213]. In endothelial cells, SIRT6 interacts with the adaptor protein ASC, thereby suppressing its acetylation. This inhibition prevents the interaction between ASC and NLRP3, resulting in the suppression of endothelial cell pyroptosis in the aortic root and the amelioration of atherosclerosis [214].

In CRC cells, histone deacetylase (HDAC) 2 is required for the suppression of NLRP3 transcription by inhibiting H3K27 acetylation-induced recruitment of the BRD4/p-p65 complex [216]. In aged mice, splenectomy under sevoflurane anesthesia increased HDAC6 expression, contributing to postoperative cognitive dysfunction by activating NLRP3-induced pyroptosis through the HSP90/HSP70 pathway in hippocampal microglia [217]. In inflammatory macrophages, the sustained expression of phosphoglycerate dehydrogenase, the rate-limiting enzyme of de novo serine synthesis, contributes to the activation of the NLRP3 inflammasome through NLRP3-K21/22/24 and ASC-K21/22/24 acetylation-mediated activation [218]. A recent study underscored the significance of glucocorticoid-induced TNFR-related enhancement of NLRP3 inflammasome activity triggered by lysophosphatidylcholine [219]. Mechanistically, glucocorticoid-induced TNFR competes with NLRP3, impeding its interaction with the E3 ligase MARCH7, which is then followed by SIRT2 degradation, eventually inhibiting ubiquitination while increasing NLRP3 acetylation [219].

Several studies have confirmed the acetylation-mediated transcription of NLRP3 in the activation of the inflammasome. For example, p300 is required for p65 acetylation, promoting the transcription of inflammatory factors and the transcription of NLRP3 [220]. Respiratory syncytial virus infection induces orosomucoid-like protein 3 (ORMDL3) through p300 histone acetylation, leading to NLRP3 inflammasome overexpression [221]. KAT2A levels are increased in the synovial tissues of patients with rheumatoid arthritis and mice with experimental arthritis [222]. KAT2A-mediated glycolysis reprogramming is mediated through the suppression of nuclear factor-erythroid 2-related factor 2 activity. Epigenetic licensing of the NLRP3 inflammasome is strongly associated with the pathogenesis of rheumatoid arthritis [222].

Recent studies have shown that protein acetylation induces the mitochondrial recruitment of ASC, which activates the NLRP3 inflammasome. In the context of high-fat diet-induced obesity, the acetylation of K255 on the α subunit of hydroxyl-CoA dehydrogenase triggers the mitochondrial localization of ASC, thereby activating the NLRP3 inflammasome and contributing to myocardial fibrosis [223]. Upregulated sphingosine kinase 2 in patients with acute respiratory distress syndrome increased nuclear sphingosine-1-phosphate levels, limiting HDAC activity and facilitating p53 acetylation [224]. p53 acetylation enhances its binding to the NLRP3 promoter, thereby increasing NLRP3 expression and activating inflammasome pathology in acute lung injury [224]. These findings suggest that fine-tuning the acetylation of NLRP3 inflammasome components is crucial for preventing acute and chronic inflammation and offers a potential drug target.

### SUMOylation of the NLRP3 inflammasome

SUMOylation involves three enzymes (E1, E2, and E3) that transfer a small Ub-like modifier (SUMO) protein to substrates, primarily facilitating noncovalent protein–protein interactions [225]. NLRP3 is SUMOylated by the E3 ligase mitochondrial-anchored protein ligase, impairing its activation, whereas sentrin/SUMO-specific protease (SENP) 6 and SENP7 reverse this effect, enhancing its activation [226]. The interaction of NLRP3 with the SUMO-conjugating enzyme UBC9 promoted the SUMOylation of NLRP3 by SUMO1, which activated NLRP3 [227]. Conversely, SENP3-mediated deSUMOylation of NLRP3 reduced the NLRP3 inflammasome activation [227]. These findings underscore the role of SENP3 in regulating NLRP3 inflammasome activity and suggest its potential as a therapeutic target for treating inflammatory diseases. Notably, the E3 SUMO ligase TRIM28 significantly increases NLRP3 expression and interacts with NLRP3 to facilitate SUMO modification, thereby inhibiting its ubiquitination and proteasomal degradation [228]. Future research is necessary to understand the role of SUMOylation in regulating each component of the NLRP3 inflammasome complex.

Ursolic acid, a natural compound with anti-inflammatory properties for treating diabetes and Parkinson’s disease, inhibits IL-1β secretion, pyroptosis, and ASC speck formation in mouse glomerular mesangial cells [229]. Mechanistically, ursolic acid enhances NLRP3 degradation by inhibiting the SUMO1-mediated SUMOylation of NLRP3 [229]. These findings provide a basis for proposing several drugs, such as ursolic acid, as potential candidates for inflammasome-related diseases.

### Citrullination of the NLRP3 inflammasome

During citrullination, peptidyl arginine deiminases (PADs) convert arginine residues in proteins into citrulline. This transformation may create neoantigens, triggering various pathological conditions, including autoimmune disorders, inflammatory diseases, and cancers [230]. However, the mechanisms by which citrullination contributes to autoimmune and autoinflammatory diseases are unclear.

Recent studies have shown that PADs are involved in the activation of the NLRP3 inflammasome complex, resulting in pathological inflammation. PAD4 is required for chromatin decondensation and promotes neutrophil extracellular trap formation in neutrophils [231]. PAD4 facilitates optimal NLRP3 inflammasome assembly by controlling ASC and NLRP3 levels posttranscriptionally [231]. Protein citrullination is prevalent in cells undergoing pyroptosis, and inhibitors of PAD activity block NLRP3 inflammasome activation. Suppressing PAD2 and PAD4 affects NLRP3 inflammasome activation and IL-1β release in macrophages [232].

Further research must elucidate the mechanisms by which citrullination influences the PTM of each NLRP3 inflammasome component and how this process contributes to the pathogenesis of autoimmune and inflammatory diseases.

### ISGylation of the NLRP3 inflammasome

A recent study revealed a novel PTM mechanism regulating the activation of the NLRP3 inflammasome involving TLR priming-mediated NLRP3 ISGylation. ISGylation, a PTM in which the interferon-stimulated gene (ISG) 15 covalently attaches to the target protein via a Ub-like enzymatic cascade that involves E1 (UBE1L) [233], E2 (UBCH8) [234], and E3 (HECT and RCC1-like domain-containing protein 5 (HERC5)) [235], has been identified as a critical step in stabilizing NLRP3 during activation [236, 237]. Notably, during SARS-CoV-2 infection and in response to type I IFNs, the expression of ISG15 and the dominant E3 ISGylation ligases HERCs is upregulated [238]. These ligases play crucial roles in NLRP3 ISGylation, which inhibits K48-linked ubiquitination and proteasomal degradation. Consequently, HERC5-mediated NLRP3 ISGylation in humans and HERC6-mediated ISGylation in mice enhances NLRP3 inflammasome activation, whereas HERC6 deficiency mitigates the NLRP3-dependent inflammation-related pathologies induced by viral infection [238]. Figure 6 presents a summary of the mechanisms involved in NLRP3 SUMOylation and ISGylation.

### Palmitoylation of the NLRP3 inflammasome

Palmitoylation, a posttranslational modification of proteins, plays an important role in the import of proteins into intracellular membranes [239, 240]. The palmitoylation reaction is mediated by a family of palmitoyl transferases known as aspartic–histidine–histidine–cysteine (DHHC) enzymes [239, 240]. Palmitoylation is relevant when NLRP3 is localized to the trans-Golgi network (TGN) during the priming phase and transiently associates with the microtubule organizing center (MTOC) during the early phase of activation [241].

Recent studies have shown that zinc finger DHHC (zDHHC) 1-mediated palmitoylation occurs sequentially at C130 (murine C126) and C958 (murine C955) of NLRP3, ultimately targeting the MTOC, which is essential for NLRP3 activation and inflammatory responses [242]. Furthermore, TLR ligation induces palmitoylation at C898 of NLRP3, leading to the translocation of NLRP3 to dispersed TGN (dTGN) vesicles, the assembly site of the inflammasome [243]. ZDHHC18-mediated palmitoylation facilitates the recruitment of NLRP3 to the MTOC in teleosts [244]. ZDHHC5 palmitoylates the LRR domain of NLRP3, promoting inflammasome assembly and activation [245]. Silencing zDHHC5 blocks NLRP3 oligomerization, the NLRP3-NEK7 interaction, and the formation of large intracellular ASC aggregates [245]. The palmitoyltransferase zDHHC17 is a key enzyme that facilitates palmitoylation at NLRP3 C419 and promotes NLRP3 activation [246]. Furthermore, zDHHC7 is essential for palmitoylation of NLRP3 at C126, promoting NLRP3 localization on the TGN and activation of the NLRP3 inflammasome complex through recruitment and oligomerization of ASC [247]. Disulfiram, an FDA-approved drug, prevents NLRP3 palmitoylation at C126 and its localization to the TGN, thereby inhibiting NLRP3 inflammasome activation [248].

Dietary phenylalanine restriction is beneficial for diabetic wounds because it reduces phenylpyruvate accumulation, which slows wound healing and stimulates inflammatory responses [249]. Phenylpyruvate uptake by macrophages in a scavenger receptor CD36-dependent manner inhibited depalmitoylase activity while increasing palmitoylation of the NLRP3 protein, thereby promoting NLRP3 inflammasome activation [249]. In contrast, zDHHC12 functions as an S-acyltransferase for NLRP3 palmitoylation, which promotes its degradation *via* the chaperone-mediated autophagy pathway [250]. *Zdhhc12* deficiency increased inflammatory symptoms in mice following alum-induced peritonitis and LPS-induced endotoxic shock [250]. Despite significant advances in identifying the molecular mechanisms underlying the palmitoylation of the NLRP3 inflammasome pathway, future studies are needed to elucidate the regulatory networks of palmitoylation with other PTMs regulating NLRP3 inflammasome activation. Furthermore, more studies will provide answers concerning how palmitoylation crosstalks with other lipid-based modifications (e.g., myristoylation and prenylation) to regulate the NLRP3 inflammasome pathway. Figure 7 summarizes palmitoylation of the NLRP3 inflammasome.

## Interactive regulation of the NLRP3 inflammasome

Numerous molecules have been shown to activate or inhibit the NLRP3 inflammasome by interacting with its components. However, how these molecules regulate the PTMs of inflammasome components is unclear. In this section, we explore both positive and negative interactive partners that modulate NLRP3 inflammasome activation within various contexts (Table 1).

**Table 1:** Positive and negative regulators of the NLRP3 inflammasome

Regulators	Binding partners	Regulation mechanisms	Consequences	Study model	Ref.
**Positive interactive partners**					
NEK7	NLRP3 (NOD and LRRs domain)	Directly targeting the promoter region of NLRP3	NLRP3 oligomerization and ASC speck formation	BMDMs, iBMDMs, HEK293T cells	[23]
	NLRP3 (LRR and HD2 domain)	Interactions between adjacent NLRP3 subunits	Caspase-1 processing and IL-1β secretion	iBMDMs	[251]
	NLRP3 (LRR domain)		NLRP3 self-association and oligomerization	iBMDMs, HEK293T cells	[253]
TXNIP	NLRP3 (LRR and NACHT)	ROS-mediated dissociation of TXNIP from the TRX	NLRP3 inflammasome activation	*Txnip*^-/-^ and *Nlrp3*^-/-^ mice, THP-1 cells	[255]
USP5	TXNIP	Stabilization of the TXNIP protein through deubiquitination	Enhancement of LPS-induced apoptosis and inflammation	Huh7 and HepG2 cells	[256]
PARP-1	TXNIP and NLRP3 (NATCH, NAD and LRR domains)	Formation of NLRP3-TXNIP complex and NLRP3 PARylation	NLRP3 inflammasome activation	*Parp-1*^-/-^ and *Nlrp3*^-/-^ BMDMs	[128]
DDX3X	NLRP3 (NBD domain)	Prevention of stress granule assembly, which inhibits ASC speck formation and pyroptosis	NLRP3 inflammasome activation	HEK293T cells, BMDMs, *Ddx3x*^*fl/fl*^ and *Ddx3x*^*fl/fl*^ *LysM*^*cre*^ mice	[257]
A_2A_R	NLRP3	Enhancement of NLRP3 inflammasome assembly and activation	Regulation of neuroinflammation in posttraumatic brain injury	Primary microglia, A_2A_R^CX3CR1^ and NLRP3^CX3CR1^ mice	[260]
UCH-L1	NLRP3 (NACHT domain)	Enhancement of NLRP3 assembly and speck formation	IL-1β production	Human macrophages and microglia	[261]
NCF4	ASC	Induction of ASC speck formation, NADPH oxidase activity, and NLRP3 inflammasome activation	Prevention of colorectal cancer progression	*Ncf4*^-/-^, *Nlrp3*^-/-^, *Aim2*^-/-^, and *Asc*^-/-^ mice	[262]
SARS-CoV-2 N protein	NLRP3	Induction of NLRP3 interaction with ASC and NLRP3 inflammasome assembly	Acceleration of lung injury and death in septic mice	*Nlrp3*^*-/-*^ C57BL/6 mice, HEK293T, A549, and THP-1 cells	[263]
PRRSV-2 nsp2	NLRP3 (NACHT domain)	IKKβ-dependent NLRP3 translocation to the dTGN facilitating ASC polymerization	Instigation of hyperinflammation due to NLRP3 inflammasome activation	HEK293T and THP-1 cells, porcine alveolar macrophages	[265]
FMDV L^pro^ protein	NLRP3 (NACHT and LRR domains)	Increased ion channels and induction of NF-κB mediated pathway	Activation of the NLRP3 inflammasome	PK-15 cells, Pigs, Pig BMDMs	[266]
HSPA13	ASC	Enhancement of ASC oligomerization and NLRP3 inflammasome activation	Inhibition of the replication of DENV and other Flaviviruses	THP-1, HEK293T, HeLa, Huh7.5, and Vero cells	[267]
ZAP	NLRP3	Activation of NLRP3 oligomerization	Induction of LPS-induced sepsis	*Zap*^+/+^ and *Zap*^-/-^ mice PMs, HEK293T cells	[268]
RACK1	NLRP3, NEK7	RACK1-mediated NLRP3–NEK7 interaction and NF-κB regulation	Regulation of *P. multocida*-induced cell death	Primary peritoneal macrophages	[269]
MIOX	NLRP3	Reduced degradation of NLRP3	Acceleration of inflammation and cardiac dysfunction	db/db mice, H9C2 cells	[270]
Negative interactive partners					
Sorcin	NLRP3	Inhibition of pyroptosis	Enhancement of tumor growth	HCC cell lines	[271]
NEDD4	NLRP3, GSDMD	Negative regulation of NLRP3, positive regulation of GSDMD	Modulation of inflammation and pyroptotic cell death	BALB/c mice, A549 cells	[272]
HECTD3	NLRP3 (NACHT and LRR domain)	Inhibition of the NLRP3–NEK7 interaction	Inhibition of NLRP3 inflammasome activation	*Hectd*^+/+^ and *Hectd*^-/-^ mice BMDMs, HEK293T cells	[273]
HAX-1	NLRP3	Inhibition of the interaction of NLRP3 and ASC	Inhibition of microglial pyroptosis and inflammatory mediator release	Microglia	[274]
PPARγ	NLRP3 (NBD and LRR domain)	Downregulation of NLRP3-ASC and NLRP3-NLRP3 interaction	Suppression of NLRP3 inflammasome activation	Leptin-deficient mice, *Pparg*^*C/-*^ and *Pparg*^*+/+*^ mice, HEK293T cells, PBMCs	[275]
FXR	NLRP3, caspase-1	Suppression of the PERK/eIF2α/CHOP pathway and decreased expression of TXNIP and NLRP3	Protection against DGR-induced gastric barrier disruption and mucosal inflammation	GES-1, RGM-1, U-937, and RAW264.7 cells	[276]
ERα	GSDMD	Inhibition of pyroptosis	Attenuation of high-fat diet-induced liver lipid steatosis	Hepatocytes	[277]

*A*_*2A*_*R* adenosine 2A receptor, *BMDMs* bone marrow-derived macrophages, *DENV* dengue virus, *DGR* duodenogastric reflux, *dTGN* dispersed trans-Golgi network, *EM* electron microscopy, *ERα* estrogen receptor α, *FMDV* foot-and-mouth disease virus, *FXR* farnesoid X receptor, *GSDMD* gasdermin D, *HAX-1* hematopoietic cell-specific protein-associated protein X1, *HCC* hepatocellular carcinoma, *HEK293* human embryonic kidney 293, *IKKβ* inhibitory kappa B kinase β, *LPS* lipopolysaccharides, *LRRs* leucine-rich repeats, *MIOX* myoinositol oxygenase, *NBD* nucleotide-binding domain, *NEDD4* neuronal precursor cell-expressed developmentally downregulated 4, *NOD* nucleotide-binding oligomerization domain, *PARP-1* poly(ADP-ribose) polymerase-1, *PARylation* poly(ADP)-ribosylation, *PBMCs* peripheral blood mononuclear cells, *P. multocida* *Pasteurella multocida*, *PMs* peritoneal macrophages, *PK-15* porcine kidney 15, *PPARγ* peroxisome proliferator-activated receptor gamma, *PRRSV-2* nsp2 porcine reproductive and respiratory syndrome virus type 2 non-structural protein 2, *RACK1* receptor for activated C kinase 1, *ROS* reactive oxygen species, *Sorcin* soluble resistance-related calcium-binding protein, *TRX* thioredoxin, *TXNIP* thioredoxin-interacting protein, *UCH-L1* ubiquitin C-terminal hydrolase 1, *USP5* ubiquitin-specific protease 5, *ZAP* zinc finger antiviral protein

### Positive interactive regulators of NLRP3 inflammasome activation

The NLRP3 inflammasome is a cytosolic multiprotein complex composed of essential components, such as NLRP3, ASC, and caspase-1; however, the structure of the NLRP3 inflammasome complex was unexplored until recently. Xiao et al. used cryogenic electron microscopy to reveal the structures of disc-shaped active NLRP3 oligomers complexed with adenosine 5′-*O*-(3-thiotriphosphate), centrosomal NEK7, and the adaptor protein ASC, which recruits caspase-1 [13]. NEK7 directly targets the NLRP3 promoter region to regulate NLRP3 assembly in a K^+^ efflux-dependent manner [23]. NEK7 forms bipartite interactions between adjacent NLRP3 subunits to activate the NLRP3 inflammasome [251]. NLRP3 variants lacking exon 5, which is stochastically regulated by alternative splicing, resulted in attenuated interactions with NEK7 [252]. A recent study revealed that in mouse macrophages, the LRR domain of NLRP3 was essential for interaction with NEK7 as well as for self-association and oligomerization [253]. However, another study reported that a direct interaction between the LRR domain of NLRP3 and NEK7 was not essential for NLRP3 inflammasome activation in human monocytes [254]. Although a specific NLRP3 residue in exon 6 of NLRP3 is required for NEK7 binding [254], exons 4, 5, 7, and 9, but not exons 6 and 8, are essential for inflammasome activation. However, a direct interaction between NEK7 and LRR was not identified in these studies, implying that some variants activate the NLRP3 inflammasome without interacting with NEK7 [254]. Notably, Xiao et al. proposed that NEK7 breaks the inactive cage, changing NLRP3 into the active NLRP3 inflammasome disc, because the C-terminal LRR domain of NLRP3 and the LRR-bound NEK7 do not engage in disc interfaces [13]. Further research is needed to determine whether NEK7 is indispensable for NLRP3 oligomerization and assembly through the licensing of the NLRP3 inflammasome.

Certain regulators have been implicated in the activation of the NLRP3 inflammasome, although their direct interaction with NLRP3 or other inflammasome components remains unclear. Over a decade ago, thioredoxin-interacting protein (TXNIP) was found to interact with NLRP3. Under inflammasome-activating conditions, TXNIP dissociates from thioredoxin and binds to NLRP3 in a ROS-dependent manner [255]. Although the mechanisms underlying its deubiquitinase activity remain elusive, USP5 has been shown to interact with TXNIP [256]. This interaction consequently facilitates NLRP3 inflammasome-associated inflammation in hepatocytes [256]. Recent research suggests that poly(ADP‒ribose) polymerase-1, which plays an important role in DNA repair, can act as a link between TXNIP and NLRP3, resulting in the formation of the NLRP3‒TXNIP complex [128]. Furthermore, poly(ADP‒ribose) polymerase-1 positively regulates NLRP3 inflammasome activation by triggering PARylation of NLRP3 in the cytosol, resulting in NLRP3 inflammasome assembly [128].

The stress granule protein DEAD-box helicase 3 X-linked (DDX3X) was shown to increase NLRP3 inflammasome activation by competing with stress granules, which reduces ASC speck formation and pyroptotic cell death in macrophages [257]. A recent study demonstrated that AKT activation reduces NLPR3 inflammasome-mediated inflammation by phosphorylating DDX3X, resulting in impaired interaction between DDX3X and NLRP3 [258]. These findings highlight the importance of DDX3X as an NLPR3-interacting component.

Previous research has shown that adenosine 2A receptor (A_2A_R)-mediated signaling maintains inflammasome activity after initial activation [259]. Further studies revealed that A_2A_R, which is widely expressed in the brain, directly interacts with NLRP3 to promote NLRP3 inflammasome assembly and activation in primary microglia, suggesting that inhibiting the interaction between A_2A_R and NLRP3 may reduce NLRP3 inflammasome assembly and activation, thereby alleviating neuroinflammation in posttraumatic brain injury [260]. Ub C-terminal hydrolase 1 interacts with the NACHT domain of NLRP3 and activates ASC oligomerization, leading to IL-1β secretion in microglia [261]. In one study, NCF4, one of the cytoplasmic subunits of NADPH oxidase, was found to work with NCF1 and NCF2 to activate the NLRP3 inflammasome by interacting with ASC. Mechanistically, the phosphorylation of membrane-bound NCF4 in the NADPH complex switched its location to the cytoplasmic region, facilitating ASC oligomerization and speck formation [262].

Several NLRP3 regulators have been implicated in various infectious conditions. A recent study revealed that the SARS-CoV-2 N protein enhances NLRP3 inflammasome activation by directly interacting with the NLRP3 protein, increasing the interaction of NLRP3 with ASCs and allowing the assembly of the NLRP3 inflammasome [263]. Suppressing the NLRP3 inflammasome reduces COVID-19-like pathology, indicating that NLRP3 inflammasome activation plays an important role in the pathogenesis of SARS-CoV-2 infection [264]. Porcine reproductive and respiratory syndrome virus type 2, a member of the *Arteriviridae* family, produces nonstructural protein 2, which interacts with the NACHT domain of NLRP3 [265]. This interaction promotes IKKβ-dependent NLRP3 translocation to the dTGN, leading to ASC polymerization and NLRP3 inflammasome activation [265]. In foot-and-mouth disease virus infection, the foot-and-mouth disease virus protein L^pro^ induces Ca^2+^ influx and K^+^ efflux, which trigger NLRP3 activation, and directly interacts with the NLRP3 NACHT and LRR domains to promote NLRP3 inflammasome assembly [266]. HSPA13, a stress chaperone that is a member of the HSP70 family, inhibits the replication of dengue virus and other flaviviruses, including Zika virus, yellow fever virus, and Japanese encephalitis virus [267]. HSPA13 interacts with ASC to promote oligomerization and NLRP3 inflammasome assembly, leading to IL-1β secretion during dengue virus infection [267]. NLRP3 activation is positively regulated by zinc finger antiviral protein, which promotes viral RNA degradation and regulates host antiviral immune responses. In peritoneal macrophages, zinc finger antiviral protein interacts with NLRP3 and promotes its oligomerization, which facilitates NLRP3 inflammasome activation [268]. Receptor for activated C kinase 1 (RACK1), a scaffold protein, regulates NF-κB and promotes NLRP3 inflammasome activation during *Pasteurella multocida* infection [269]. Immunofluorescence staining and immunoprecipitation studies revealed that RACK1 interacts with NLRP3 and NEK7 and that inhibiting K^+^ efflux significantly attenuates the RACK1–NLRP3–NEK7 interaction [269]. Myoinositol oxygenase expedited the activation of the NLRP3 inflammasome, contributing to cardiac dysfunction in infection-induced cardiac dysfunction models [270]. Myoinositol oxygenase-induced inflammation ensues from its interaction with NLRP3, impeding its degradation [270]. Studies must determine whether these proteins that are involved in specific infectious conditions universally regulate NLRP3 inflammasome components and influence inflammatory responses in other disease contexts.

### Negative interactive regulators of NLRP3 inflammasome activation

Several studies have demonstrated the presence of negative regulators or pathways involved in NLRP3 inflammasome activation. Sorcin—a soluble, resistance-related calcium-binding protein—is highly expressed in hepatocellular carcinoma tissues and cells. Sorcin interacts with the NLRP3 inflammasome to inhibit pyroptosis in hepatocellular carcinoma cells, resulting in cell proliferation, migration, and invasion [271]. During coinfection with influenza virus and *Streptococcus pneumoniae*, the E3 Ub ligase neuronal precursor cell-expressed developmentally downregulated 4 (NEDD4) interacts directly with NLRP3 and GSDMD. NEDD4 has a negative regulatory effect on NLRP3 but a positive effect on GSDMD. The specific role of NEDD4 in regulating the NLRP3 inflammasome and GSDMD-mediated pyroptosis signaling must be validated [272].

Other studies have shown that negative regulators hinder the interaction of NLRP3 with other elements, such as NEK7, ASC, or caspase-1. For example, the E3 Ub ligase HECTD3, regardless of its E3 ligase activity, interacts with the NACHT/LRR domain of NLRP3 through its DOC domain [273]. This interaction blocks the NLRP3–NEK7 interaction and consequently inhibits NLRP3 inflammasome activation [273]. HAX-1, a prosurvival protein, is important in cerebrovascular diseases, such as acute cerebral ischemia. A recent study revealed that after cerebral IR injury, HAX-1 expression decreased, whereas pyroptosis increased. HAX-1 can inhibit microglial pyroptosis both in vivo and in vitro, partially by inhibiting the interaction of NLRP3 and ASC through competitive binding [274]. Peroxisome proliferator-activated receptor gamma (PPARγ), an energy metabolism regulator, plays an anti-inflammatory role by inhibiting NLRP3 activation. PPARγ prevents NLRP3 inflammasome formation by decreasing NLRP3–ASC and NLRP3–NLRP3 interactions as well as NLRP3-dependent ASC oligomerization [275]. Mechanistically, the PPARγ DNA-binding domain interacts with the NLRP3 NBD and LRR domains [275]. Another negative regulator is the farnesoid X receptor (FXR), which inhibits the duodenogastric reflux-induced NLRP3 inflammasome and pyroptosis by physically interacting with NLRP3 and caspase-1 [276]. FXR reduces TXNIP and NLRP3 expression and ER stress through the PERK/eIF2α/CHOP pathway [276].

Estrogen receptor α inhibits NLRP3 inflammasome activation, GSDMD N-terminal fragment generation, and the secretion of proinflammatory cytokines, including IL-1β and IL-18 [277]. Mechanistically, estrogen receptor α inhibits hepatocyte pyroptosis by directly interacting with GSDMD [277]. These findings highlight the potential of targeting these interactive partners for the development of therapeutic strategies. Further research is warranted to elucidate other interactive partners of NLRP3 that negatively regulate its function. Such insights will be crucial in developing strategies to address immune disorders resulting from NLRP3 inflammasome overactivation.

## Spatiotemporal and sequential regulation of the NLRP3 inflammasome

**Fig. 8:**
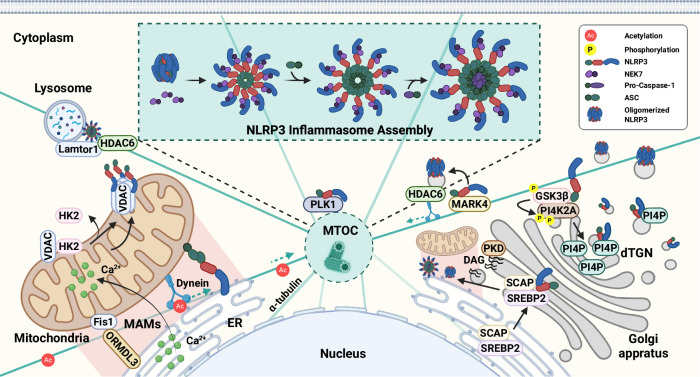
NLRP3 inflammasome activation is tightly regulated by subcellular localization dynamics. MAMs serve as key signaling hubs for NLRP3 assembly by promoting ER-mitochondria tethering and local calcium flux. Mitochondrial ASC binds to NLRP3 on the ER through acetylated α-tubulin- and dynein-dependent mitochondrial transport, facilitating the formation of MAMs. ER-mitochondria contacts mediated by ORMDL3 and Fis1, as well as Ca²⁺ flux via VDAC oligomerization (triggered by HK2 dissociation), further promote NLRP3 assembly. NEK7 and PLK1, located at the MTOC, are also involved in NLRP3 inflammasome activation. NLRP3 recruited to the dTGN can be transported to the MTOC by HDAC6 and MARK4. Furthermore, NLRP3 interacts with SREBP2 and SCAP to form a ternary complex, which translocates to the Golgi apparatus near the mitochondria, facilitating optimal inflammasome assembly and activation of the NLRP3 inflammasome. PKD, a key effector of DAG, induces MAMs to localize near Golgi membranes, facilitating NLRP3 oligomerization and activation. Activated GSK3β phosphorylates PI4K2A, enhancing PI4P production at the TGN, which facilitates NLRP3 localization and oligomerization. HDAC6 enhances the binding of NLRP3 to Lamtor1 located on the lysosome, leading to NLRP3 inflammasome activation. Ac acetylated, Ca calcium, DAG diacylglycerol, dTGN dispersed trans-Golgi network, ER endoplasmic reticulum, Fis1 fission 1, GSK3β glycogen synthase kinase 3β, HDAC6 dynein adaptor histone deacetylase 6, HK2 hexokinase 2, MAM mitochondria-associated membrane, MARK4 microtubule-affinity regulating kinase 4, MTOC microtubule-organizing center, NEK7 NIMA-related kinase 7, ORMDL3 orosomucoid-like protein 3, PI4K2A phosphatidylinositol 4-kinase type 2 alpha, PKD protein kinase D, PLK1 polo-like kinase 1, SCAP SREBP cleavage-activating protein, SREBP sterol regulatory element binding protein, VDAC voltage-dependent anion channel

Recent structural and functional studies have demonstrated that the NLRP3 inflammasome complex is facilitated by spatiotemporal interactions of inflammasome components at organelle contact sites, which begin at the MTOC and complex at the ER–Golgi boundaries. The current understanding is a sequential structural formation from the inactive NLRP3 cage, the NLRP3 disk containing NEK7–ASC, and PYD–PYD and CARD–CARD homotypic filamentous inflammasome scaffolds. The processes are summarized in detail in recent reviews [2, 7]. In the following section, we briefly investigate recent findings that reveal the key molecules and mechanistic processes that mediate the spatiotemporal regulation of the NLRP3 inflammasome complex (Fig. 8).

### ER–mitochondrion interaction and the MTOC

Studies have reported that the NLRP3 inflammasome complex assembles in mitochondria-associated ER membranes (MAMs), which are specialized junctions that connect the ER and mitochondria [278]. Acetylated α-tubulin and dynein-dependent mitochondrial transport are required for the formation of MAMs and the assembly of mitochondrial ASC to NLRP3 localized at the ER [278]. In addition to known NLRP3 signals, chronic social defeat stress triggers the modification of MAMs, where the formation of the inositol 1,2,3-triphosphate receptor 3–glucose-regulated protein 75–VDAC1 complex leads to NLRP3 inflammasome aggregation and activation in microglia [279]. Several molecules are proposed to mediate the contact between the ER and mitochondria. For example, orosomucoid-like protein 3 overexpression increases mitochondrial fragmentation and ER contacts, allowing NLRP3 inflammasome activation [280]. Orosomucoid-like protein 3 is found not only in the ER but also in mitochondria and MAMs during inflammation, where it interacts with mitochondrial fission 1, a key regulator of mitochondrial dynamics [280]. Additionally, VDAC plays an important role in NLRP3 inflammasome assembly and activation [281]. Mechanistically, the dissociation of hexokinase 2 from VDAC induces VDAC oligomerization through calcium transport from the ER to mitochondria, allowing VDAC oligomers to aggregate with NLRP3 during the initial assembly of the NLRP3 inflammasome complex [281].

Recent studies have revealed that optimal signal transduction sites through microtubules are crucial for the effective and complete activation of the NLRP3 inflammasome [6]. Notably, microtubule-affinity regulating kinase 4, a serine/threonine kinase family member, binds to NLRP3 and drives it to the MTOC, triggering inflammasome speck formation and assembly [282]. During this process, the interaction of NEK7 with NLRP3 is localized to the MTOC, which facilitates the assembly of the NLRP3 inflammasome [242]. In addition, polo-like kinase 1, a key regulator of mitosis initiation, coordinates the MTOC structure and NLRP3 subcellular positioning in response to NLRP3 inflammasome activation during cell interphase [283]. Recent advances in cryoelectron microscopy have revealed that the inactive octameric structure of NLRP3 is disrupted and interacts with NEK7 to form a disk-like assembled structure consisting of NEK7–NLRP3 units [284]. This disk-like structure functions as a protein complex platform to recruit other inflammasome components, such as ASC and pro-caspase-1 [284]. Thus, the communication between multiple components at MTOCs may lead to the assembly and activation of the NLRP3 inflammasome complex at ER–mitochondrial boundaries. A deeper understanding of the dynamic activation of NLRP3 inflammasome assembly can provide potential targeted therapeutic strategies for interrupting the spatial communication of the NLRP3 inflammasome.

### Trans-Golgi network

Recent studies have shown that the Golgi apparatus functions as a scaffold for NLRP3 aggregation and activation of NLRP3 inflammasome assembly. Under NLRP3 stimulation, the TGN disassembles into the dTGN. NLRP3 is recruited to the dTGN and interacts with phosphatidylinositol-4-phosphate, thereby leading to NLRP3 aggregation and ASC polymerization and the caspase-1 signaling cascade [285]. A recent study revealed that modified NLRP3, where *S*-acylation occurs at C130, is essential for its translocation to the Golgi apparatus to further assemble the NLRP3 inflammasome complex [286]. Moreover, NLRP3 forms a ternary complex with SREBP2 and SCAP and translocates to the Golgi apparatus adjacent to the mitochondria for optimal inflammasome assembly and NLRP3 inflammasome activation [50]. Notably, NLRP3 inflammasome stimuli induce MAMs to localize near Golgi membranes through Golgi-mediated protein kinase D signaling, which facilitates NLRP3 oligomerization and activation [146]. Recent findings have shown that glycogen synthase kinase 3β binds to NLRP3 and recruits it to mitochondria before it transitions to the TGN through a temporal interaction [287]. During this process, the activation of glycogen synthase kinase 3β results in the phosphorylation of phosphatidylinositol 4-kinase type 2α in the TGN, leading to extended NLRP3 oligomerization [287]. These findings indicate that multiple signaling players are involved in the interplay between spatial dynamics, NLRP3 aggregation, and inflammasome assembly. However, whether the interorganellar interactions among the dTGN, MAM, and MTOC are sequential, simultaneous, or context dependent is unclear.

The dynein adaptor HDAC6 was found to be necessary for microtubule transport and inflammasome assembly at the MTOC [288]. Under HDAC6 deficiency, NLRP3 is trapped at the TGN, indicating that HDAC6 is crucial for the transport of NLRP3 to the MTOC through microtubules [288]. Lamtor1, a key component of the Ragulator complex expressed on the lysosomal membrane, can bind both NLRP3 and HDAC6 [289]. As a result, HDAC6 activated the NLRP3 inflammasome by enhancing the Lamtor1–NLRP3 interaction [289]. These findings suggest that a more intricate regulatory mechanism, potentially involving epigenetic control, contributes to the coordination of multiorganelle interactions among the MTOC, dTGN, and even lysosomes to establish the NLRP3 inflammasome complex. Future research must uncover how organelles orchestrate the transport of inflammasome components to focal assembly sites. To achieve this goal, emerging advanced tools, such as superresolution microscopy, organelle-specific biosensors, and sophisticated biochemical approaches, will be crucial to provide real-time insights during NLRP3 inflammasome assembly. Such efforts will not only illuminate the biochemical pathways underlying inflammasome signaling but also integrate interorganelle communication and architecture, thereby mapping inflammasome assembly in detail. Understanding the spatial cues and elucidating the undefined compartments for spatiotemporal coordination could lead to innovative therapeutic approaches for targeted interventions aimed at modulating NLRP3-related pathologies.

## Noncoding RNA-mediated regulation of the NLRP3 inflammasome

Noncoding RNAs (ncRNAs), which lack protein-coding capacity, play pivotal roles in cellular and molecular processes, particularly in gene transcription, translation regulation, and epigenetic modulation. These include microRNAs (miRNAs) and piwi-interacting RNAs, which are small ncRNAs with <200 nucleotides, and long intergenic ncRNAs (lincRNAs) and natural antisense transcripts, which have ≥200 nucleotides [290]. Increasing evidence suggests the involvement of ncRNAs in modulating the NLRP3 inflammasome, influencing both the transcription and PTM regulation of essential components and associated factors in its activation [291]. In this section, we briefly outline recent advancements in understanding how ncRNAs regulate the NLRP3 inflammasome and discuss their implications in pathological contexts.

A newly discovered function of LncZFAS1 has emerged, demonstrating its role in negatively regulating NLRP3 inflammasome activation and pyroptosis in human neuroblast cells treated with 1-methyl-4-phenylpyridinium, serving as an in vitro model of Parkinson’s disease [292]. Mechanistically, LncZFAS1 directly interferes with miR590-3p and promotes the ubiquitination of TXNIP by the MIB1 E3 Ub ligase, thereby modulating NLRP3 inflammasome activation in neuroblasts [292]. In contrast, differential expression of the lncRNA colorectal neoplasia exacerbates increased activation of the NLRP3 inflammasome, thus worsening the progression of IgA nephropathy [293]. Inhibiting the differential expression of colorectal neoplasia mitigated the inflammatory responses linked to the NLRP3 inflammasome by regulating the level of the NLRP3 protein and facilitating its ubiquitination and degradation in macrophages [293].

miR-369-3p targets BRCC3 to downregulate the expression of NLRP3, consequently suppressing its activation [294]. This inhibition extends to the maturation of pro-IL-1β and pro-IL-18 [294]. A recent study revealed that the lncRNA growth-arrest specific transcript 5 (GAS5) plays a protective role in nonalcoholic fatty liver disease by acting as a sponge for miR-28a-5p, which subsequently targets MARCH7 [295]. This mechanism helps ameliorate NLRP3 inflammasome-mediated pyroptosis in both in vivo and in vitro models of nonalcoholic fatty liver disease [295]. Furthermore, GAS5 can suppress caspase-1 activity, NLRP3 inflammasome activation, and pyroptosis in cardiac muscle cells treated with high glucose [296]. This effect is facilitated by the interaction between GAS5 and miR-34b-3p, resulting in the downregulation of aryl hydrocarbon receptor expression and ultimately improving diabetic cardiomyopathy [296]. These findings underscore the potential of GAS5-mediated regulation of miRNAs, which target proteins involved in NLRP3 inflammasome activation, as promising biomarkers for inflammatory and metabolic disorders.

Specific protein 1-induced miR-144-3p targets PTEN expression, enhancing IL-1β-induced pyroptosis in chondrocytes while inhibiting the activation of the PTEN-induced kinase 1 (PINK1)/parkin signaling pathway [297]. This finding has potential therapeutic implications for the treatment of rheumatoid arthritis [297]. Furthermore, miR-146a-5p targets RING finger protein 8, modulating Notch1/mTORC1 signaling and NLRP3 inflammasome activation in intestinal epithelial cells, such as Caco-2/HT-29 cells [298]. In myocardial tissues and cardiomyocytes, miR-96-5p targets NLRP3 to regulate sepsis-induced myocardial injury [299]. Mechanistically, USP7 upregulates the transcription factor sex-determining region Y-box 9, suppresses miR-96-5p and modulates NLRP3 inflammasome activation and pyroptosis [299]. These findings underscore the roles of ncRNAs in regulating molecules involved in the NLRP3 inflammasome pathway, emphasizing the importance of investigating their identification and functional roles in disease pathologies.

Circular RNAs (circRNAs), characterized by their closed-loop structure, have been implicated in the regulation of NLRP3 inflammasome activation in several disease models. Notably, patients with ulcerative colitis presented decreased levels of autophagy, circRNA HECTD1, and human antigen R [300]. In colonic epithelial cells, elevated levels of the circRNA HECTD1 or human antigen R are correlated with reduced inflammation and increased autophagy [300]. Another circRNA, hsa_circ_0043621, contributes to the pathogenesis of atherosclerosis by acting as a molecular sponge for miR-223-3p, increasing NLRP3 expression [301]. Furthermore, the circRNA circVmn2r1 targeted miR-223-3p to accelerate kidney aging by upregulating NLRP3 expression in a mouse model [302]. Exploring novel ncRNAs involved in regulating NLRP3 inflammasome components and the mechanisms may reveal therapeutic strategies for treating inflammasome-associated diseases.

## Other: mRNA stability, autophagy, and unknown

### Immunometabolic regulation of the NLRP3 inflammasome

Distinct immunometabolic pathways, including glycolysis, oxidative phosphorylation, and lipid metabolism, may influence NLRP3 inflammasome activation. However, the precise profiles of immunometabolic pathways involved in the regulation of NLRP3 inflammasome activation are unclear. A correlation between the activation of the glycolytic pathway and NLRP3 inflammasome activation has been suggested, although it is unclear. An early study revealed that pyruvate kinase M2 promotes NLRP3 and AIM2 inflammasome activation by regulating the phosphorylation of the protein kinase eukaryotic translation initiation factor 2 alpha kinase 2 [303]. A recent study revealed that the glycolytic enzyme aldolase A (ALDOA), which is pivotal for monitoring glycolytic flux, is essential for NLRP3 inflammasome activation [304]. Furthermore, ALDOA modulates this process by suppressing p62/SQSTM1, an essential receptor for mitophagy, thereby facilitating NLRP3 inflammasome activation [304]. Conversely, inhibiting ALDOA triggered the activation of parkin-mediated mitophagy and the subsequent elimination of damaged mitochondria through the AMP-activated protein kinase (AMPK)–forkhead box protein O3 pathway [304].

The suppression of pyruvate dehydrogenase kinase inhibited the NLRP3 inflammasome, reversed immunometabolic reprogramming, promoted autophagy, and preserved mitochondrial function [305]. However, these effects do not depend on the canonical function of pyruvate dehydrogenase kinase [305].

The crucial metabolic sensor AMPK can be a target of NLRP3 activators. A recent study showed that amyloid-β triggered NLRP3 inflammasome activation in microglia by regulating AMPK, thereby dysregulating metabolism [306]. Several studies have suggested the role of individual immunometabolites in the regulation of NLRP3 inflammasome activation. For example, itaconate suppressed NLRP3 inflammasome activation by disrupting the interaction between NEK7 and NLRP3 [307]. A recent study revealed the regulatory role of fructose in suppressing NLRP3 inflammasome activation and M1-like macrophage polarization by inducing the interaction between hexokinase 2 and inositol 1,4,5-triphosphate receptor type 3 [308]. This action of fructose promotes CRC tumorigenesis [308]. Another study revealed the role of NLRP3 in modulating the mitochondrial and metabolic functions that influence the progression of Alzheimer’s disease [309]. The loss of NLRP3-regulated microglial metabolism increases glutamine use and α-ketoglutarate levels, resulting in cellular reprogramming that facilitates amyloid-β peptide clearance, epigenetic modifications, and changes in gene transcription [309]. Future studies are warranted to elucidate the molecular and epigenetic mechanisms by which distinct metabolic pathways, enzymes, or metabolites regulate NLRP3 inflammasome activation. Additionally, the roles and mechanisms of specific immunometabolites should be investigated in the context of the NLRP3 inflammasome-related pathogenesis of disease conditions, such as cancer.

### mRNA stability

METTL3, a m^6^A methyltransferase, has been reported to be important for pyroptosis and M1 polarization of hepatic macrophages, thus contributing to liver fibrosis [310]. Mechanistically, METTL3 facilitates the degradation of USP8 mRNA through m^6^A methylation and stabilizes metastasis-associated lung adenocarcinoma transcript 1, a highly m^6^A-modified lncRNA, through interaction with polypyrimidine tract-binding protein 1 [310]. This regulation further influences TAK1 signaling, which is essential for NF-κB activation via the phosphorylation of downstream IKKs [311], thereby facilitating pyroptosis and inflammation in hepatic macrophages [310]. Furthermore, METTL3–METTL14 complex-mediated m^6^A modification reduces A20 stability, which diminishes NEK7 ubiquitination and subsequent activation of NLRP3 inflammasome assembly [202]. Ub C-terminal hydrolase L5, which is modified and stabilized by the METTL14/YTHDF1 axis through m^6^A, promotes NLRP3 inflammasome activation by deubiquitinating NLRP3. This mechanism contributes to vascular remodeling and inflammation in atherosclerosis [312].

A recent study revealed that fat mass and obesity-associated proteins reduce NLRP3-triggered pyroptosis and inflammasome activation by destabilizing Cbl proto-oncogene mRNA through the inhibition of m^6^A modification [313]. This obesity-associated protein-mediated suppression of pyroptosis mitigated myocardial IR injury by inhibiting Cbl-induced β-catenin ubiquitination and degradation [313].

### YAP/β-catenin pathway

Reduced expression of KAT5 and stress-induced phosphoprotein 1 homology and U-box-containing protein 1, an E3 Ub ligase, in addition to elevated levels of large tumor suppressor kinase 2 (LATS2), is correlated with pyroptosis in cardiomyocytes and contributes to the pathogenesis of myocardial IR injury related to NLRP3-mediated pyroptosis [314]. Mechanistically, KAT5 promoted the transcription of U-box-containing protein 1 by modulating the acetylation and subsequent degradation of LATS2, which led to YAP/β-catenin signaling. Therefore, the overexpression of KAT5 or silencing of LATS2 attenuated cardiomyocyte pyroptosis through the activation of the YAP/β-catenin pathway [314].

### ER stress

ER stress is a physiological response for protein homeostasis through the activation of the unfolded protein response, although severe and persistent ER stress is often detrimental and related to pathological conditions [315, 316]. MAMs are believed to be critical platforms for physiological responses and the activation of NLRP3 inflammasome complex formation [317, 318]. Emerging evidence suggests that dysregulated or excessive ER stress is linked to the activation of the NLRP3 inflammasome, resulting in the pathologies of inflammatory diseases [316], such as diabetes mellitus [319], inflammatory bowel disease [320], chronic liver disease [321], rheumatic disease [322], and neurodegenerative disease [323].

The RNase activity of inositol-requiring enzyme 1α, a key factor of the unfolded protein response, is required for the structural assembly of the NLRP3 inflammasome complex and IL-1β secretion [324]. In *S. pneumoniae*-infected cells, an ER stress factor, activating transcription factor-3, is involved in NLRP3 inflammasome activation and IL-1β secretion [325]. In osteoblasts, DNA damage-inducible transcript 3/CCAAT/enhancer-binding protein homologous protein, a key player in ER stress, mediates inflammasome-associated pyroptotic cell death through the suppression of mitophagy [326]. Recent studies have revealed the causal role of TXNIP, which interacts with thioredoxin and inhibits antioxidant function, in the activation of ER stress-induced NLRP3 inflammasome complex formation in the context of various pathologies [327–329].

Several molecules involved in the regulation of ER stress are believed to negatively regulate NLRP3 inflammasome activation. For example, FXR activation contributes to the negative regulation of ER stress-mediated activation of the NLRP3 inflammasome to attenuate hepatocyte death and hepatic injury [330]. Sestrin2, a stress-inducible protein, plays a role in suppresing ER stress, as well as NLRP3 inflammasome activation and pyroptosis, during cholestatic liver injury [331]. However, it is unclear whether Sestrin2 deficiency-induced ER stress is linked to NLRP3 inflammasome activation, thereby contributing to hepatic pathologies.

### Autophagy

Although our review does not extensively explore the roles of autophagy in regulating the NLRP3 inflammasome, we highlight recent studies that emphasize specific autophagy pathways and components involved in this regulation. In an acute pancreatitis model, the mitophagy kinases PINK1 and parkin play crucial roles in suppressing NLRP3 inflammasome activation, thereby mitigating inflammatory cell infiltration and pancreatic damage [332]. PINK1 deficiency enhances NLRP3 inflammasome activation and promotes the carcinogenesis of CRC, at least partly through increased mitochondrial iron transporters via the upregulation of cellular and mitochondrial iron levels [333]. Moreover, blockade of mitochondrial iron and superoxide levels by deferiprone and minocycline is beneficial for the treatment of colon cancers [333], suggesting the potential of therapeutics targeting the mitophagy–inflammasome axis. The E3 ligase parkin, which is vital for mitophagy activation, mitigated glutamate-induced excessive NLRP3 inflammasome activation, mitochondrial dysfunction, and oxidative stress, thereby exerting neuroprotective effects in an acute optic nerve injury model [334].

Several key molecules possibly contribute to the autophagic regulation of NLRP3 inflammasome activation. USP5 negatively regulates NLRP3 inflammasome activation by inducing the autophagic degradation of NLRP3 [335]. This function involves the K48-linked polyubiquitination of NLRP3, facilitating its degradation through the autophagy–lysosomal pathway, independent of its DUB activity. USP5 recruits the E3 ligase MARCH7 as a key scaffold to coordinate the autophagic degradation of NLRP3, suggesting therapeutic potential for the inflammatory innate immune response [335]. α/β-hydrolase domain-containing 8 negatively regulates inflammasome activation by interacting with NLRP3 for its degradation through chaperone-mediated autophagy [336]. Moreover, cyclin-dependent kinase 5 regulatory subunit-associated protein 3, a substrate of the E3 ligase, is critical for autophagolysosome degradation, thus inhibiting *S. agalactiae*-induced inflammation through the suppression of the NLRP3 inflammasome and pyroptotic cell death [337]. Recent research has suggested that chronic sleep deprivation causes gut microbiota dysbiosis, which in turn activates glycogen synthase kinase-3 beta in the brain [338]. Activated glycogen synthase kinase-3 beta is a key regulator of NLRP3-mediated autophagy dysfunction, facilitating tau hyperphosphorylation, which leads to cognitive impairment [338].

MafB, an MAF transcription factor with binding affinity for a specific DNA element motif [339], negatively regulates the NLRP3 inflammasome by sustaining p62 levels through the induction of mitophagy and mtROS production [340]. A deficiency in MafB led to increased systemic and lung inflammation as well as defective clearance of *Pseudomonas aeruginosa* in vivo [340]. These findings demonstrate that several autophagic mediators may represent potential therapeutic targets in conditions associated with altered NLRP3 inflammasome activation. Future studies are needed to elucidate the molecular interaction between autophagy and the inflammasome to provide insights for developing autophagy-based treatments for NLRP3-related inflammatory and autoimmune diseases.

## Brief overview of NLRP3-targeted therapeutics for inflammatory diseases

Previous studies have identified the importance of NLRP3 in rare autoinflammatory syndromes, collectively termed CAPS, in which mutations in the NLRP3 gene cause a group of inherited autoinflammatory disorders [341, 342]. Within this spectrum, three primary conditions have been recognized: familial cold autoinflammatory syndrome (FCAS), Muckle–Wells syndrome (MWS), and neonatal-onset multisystem inflammatory disease (NOMID), also known as chronic infantile neurological cutaneous and articular syndrome (CINCA). These diseases vary in severity but share a common pathophysiology, which involves excessive production of IL-1β due to hyperactivation of the NLRP3 inflammasome [343, 344]. FCAS, a milder form of CAPS, is triggered by cold exposure and presents with fever, rash, and joint pain that usually resolve once the inciting stimulus is removed [341]. MWS presents with similar symptoms in addition to the risk of progressive hearing loss and renal amyloidosis [341]. NOMID/CINCA, the most severe CAPS form, appears at or shortly after birth and features persistent systemic inflammation, distinctive neurological involvement, and a significant risk of joint deformities. If left untreated, NOMID/CINCA can lead to profound developmental impairment and life-threatening complications [342, 345]. Therefore, genetic mutations in the NLRP3 inflammasome are key drivers of autoinflammatory syndromes.

In addition to inherited autoinflammatory diseases, dysregulated NLRP3 activation is associated with various human diseases characterized by chronic inflammation, degeneration, and cancer [346, 347]. NLRP3-driven production of IL-1β and IL-18 contributes to the tissue damage observed in various autoimmune and autoinflammatory diseases, such as gout, rheumatoid arthritis, type 2 diabetes mellitus, cardiovascular disorders, and infectious diseases [348–352]. Monosodium urate crystals in gout and cholesterol crystals in atherosclerosis directly activate the NLRP3 inflammasome, which intensifies local inflammation [348, 351]. Chronic low-grade inflammation in obesity and type 2 diabetes mellitus activates the NLRP3 inflammasome *via* metabolic stressors, leading to insulin resistance [350, 353]. Furthermore, in neurodegenerative disorders, such as Alzheimer’s disease and Parkinson’s disease, protein aggregates (e.g., amyloid-β and α-synuclein) and mitochondrial dysfunction induce inflammasome activation, aggravating neuroinflammation and neuronal injury [309, 354, 355]. Several extensive reviews have discussed NLRP3 inflammasome activation, its pathological contributions to various disease models, and therapeutic strategies targeting NLRP3, ASC, and other inflammasome components and approaches for regulating pyroptosis [346, 347, 356, 357]. Therefore, this section explores selected examples of therapeutic candidates targeting NLRP3, focusing on their mechanisms and disease models in which they have been studied. We emphasize only a subset of drug development strategies because of space limitations. Table 2 summarizes the currently identified NLRP3-targeted therapies, categorized according to their mode of inhibition (i.e., direct and indirect NLRP3 inhibitors) and IL-1β blockade.

**Table 2:** Therapies targeting NLRP3-driven inflammation

Type	Characteristics	Target diseases	Reference
**Direct NLRP3 inhibitors**			
MCC950 (CRID3)	Binds to the NACHT domain	CAPS, MWS, MS, Alzheimer’s disease, HF, IBD	[358–364]
OLT1177 (Dapansutrile)	Binds to the NACHT domain	CAPS, gout, HF, MS	[365–368]
NP3-562	Binds to the NACHT domain	-	[369]
GDC-2394	Binds to the NACHT domain	-	[370, 371]
CY-09	Binds to the ATP-binding motif of the NACHT domain	CAPS, type 2 DM, gout	[372]
ZYIL1 (Usnoflast)	Inhibit the NLRP3 inflammasome	CAPS, Parkinson’s disease	[373, 374]
Compound A	Binds to the NACHT domain	CAPS	[375]
RRx-001	Covalently modifies C409 in the NACHT domain Inhibits NF-κB and induces Nrf2 Penetrates the blood-brain barrier	IBD, MS	[376]
Oridonin	Covalently modifies C279 in the NACHT domain	Peritonitis, gout, type 2 DM	[377]
4-octyl itaconate	Dicarboxypropylates C548 in the NACHT domain	CAPS, eosinophilic asthma	[307, 378]
**Indirect NLRP3 modulators**			
Glyburide	Inhibits ATP-sensitive K^+^ channels Reduces ASC aggregation and IL-1β secretion	Endotoxemia	[379]
BHB	Inhibits K^+^ efflux, ASC oligomerization, and IL-1β and IL-18 release	CAPS, Alzheimer’s disease, spinal cord injury	[382–385]
Dimethyl fumarate	Inhibits mtROS	Psoriasis, MS, auto-immune hepatitis	[386–388]
Rasagiline	Suppresses mtROS production	Parkinson’s disease	[389]
Metformin	Blocks mtDNA synthesis and cytosolic mtDNA release	SARS-CoV-2-induced acute respiratory distress syndrome	[390]
Nickel–cobalt alloy nanocrystals	Inhibits Inflammasome assembly by downregulation of lncRNA Neat1	IBD	[391]
**IL-1 blockade therapy**			
Anakinra	Blocks both IL-1α and IL-1β	CAPS, gout, Still’s disease, Behcet’s disease-related uveitis, rheumatoid arthritis, DM, HF	[392, 394–396, 404]
Rilonacept	Sequesters IL-1α and IL-1β Has a longer half-life than anakinra	Deficiency of IL-1 receptor antagonist, CAPS, recurrent pericarditis	[397–400]
Canakinumab	Targets IL-1β	CAPS, rheumatoid arthritis, Still’s disease, Behcet’s disease-related uveitis, Schnitzler’s syndrome, atherosclerotic disease, DM	[399, 401–409]

*ASC* apoptosis-associated speck-like protein containing a caspase-recruitment domain, *ATP* adenosine triphosphate, *BHB* β-hydroxybutyrate, *CAPS* cryopyrin-associated periodic syndrome, *DM* diabetes mellitus, *HF* heart failure, *IBD* inflammatory bowel disease, *IL* interleukin, *lncRNA* long non-coding RNA, *MS* multiple sclerosis, *mtROS* mitochondrial ROS, *MWS* Muckle-Wells syndrome, *NLRP3* NACHT-, leucine-rich-repeat-, and pyrin domain-containing protein 3, *ROS* reactive oxygen species

### Direct NLRP3 inhibitors

Several small-molecule NLRP3 direct inhibitors are in preclinical or early clinical development and are designed to bind to the NACHT domain and interfere with the conformational changes or oligomerization steps required for inflammasome assembly [347]. MCC950 (also known as CP-456, 773, or CRID3) was identified as a prototype inflammasome inhibitor through its binding to the NACHT domain, preventing ATP hydrolysis and blocking the NLRP3 conformational change for activation [358–360]. In experimental models of autoimmune, neurodegenerative, and metabolic disorders, MCC950 strongly suppresses IL-1β production and alleviates disease symptoms [359, 361–364]. OLT1177 (dapansutrile) has subsequently been reported to be an inhibitor of the NLRP3 ATPase [365] and has demonstrated safety and efficacy in reducing IL-1-mediated inflammation in conditions such as gout, heart failure, and autoimmune encephalomyelitis [366–368]. NP3-562 is a potent NLRP3 inhibitor that binds to the NACHT domain of NLRP3. In preclinical studies using a mouse peritonitis model, NP3-562 decreased IL-1β release, further demonstrating its therapeutic relevance [369]. Furthermore, GDC-2394 is a potent and selective NLRP3 inhibitor that directly binds to the NACHT domain of NLRP3 [370]. However, safety concerns emerged during the first-in-human phase I clinical trial, which posed a significant challenge to its development and necessitated further modifications for its druggable development [371]. CY-09 has been reported to bind to the ATP-binding motif of the NACHT domain and inhibit ATPase activity, resulting in inflammasome assembly inhibition [372]. Preclinical studies of CY-09 have shown its benefits in type 2 diabetes mellitus models by improving insulin resistance and preserving pancreatic beta-cell function and in gout models by reducing inflammation triggered by urate crystals [372]. ZYIL1 (Usnoflast) is a selective NLRP3 inflammasome inhibitor that has been shown to decrease CAPS disease activity and improve symptoms in a mouse model of Parkinson’s disease [373, 374]. Recently, compound A, an MCC950-like diaryl sulfonylurea-containing novel compound, was reported to have a therapeutic effect on CAPS [375]. Multiple NLRP3 inhibitors are being evaluated in clinical trials for their safety and efficacy [346, 357].

Moreover, inhibitors that covalently modify NLRP3 and hinder its interaction with other proteins for inflammasome activation have been identified. RRx-001 interacts with C409 of NLRP3 and suppresses the interaction of NLRP3 with NEK7, thereby inhibiting inflammasome assembly and alleviating symptoms in various disease models, including systemic inflammation, colitis, and experimental autoimmune encephalomyelitis in mice [376]. Oridonin from the herb *Rabdosia rubescens* forms a covalent bond with C279 of NLRP3 and hinders the interaction of NLRP3 with NEK7. It has also shown beneficial effects against peritonitis, gout, and type 2 diabetes mellitus [377]. Furthermore, the itaconate derivative 4-octyl itaconate alkylates C548 of NLRP3, thereby inhibiting the interaction between NLRP3 and NEK7 and abrogating NLRP3 inflammasome activation while demonstrating potential therapeutic benefits in CAPS and eosinophilic asthma [307, 378]. Collectively, these direct NLRP3 inhibitors hold promise for broader therapeutic applications and potentially fewer off-target effects than other candidates that target indirect pathways and/or upstream and downstream regulators of the NLRP3 inflammasome complex. However, robust clinical data are necessary to fully establish the long-term safety, efficacy, and application of these drugs in human diseases.

### Indirect NLRP3 inhibitors

In addition to directly targeting NLRP3, the potential activities of numerous reagents and small molecules that regulate the signaling pathways upstream of NLRP3 inflammasome activation have been explored. Because a detailed investigation of these agents is beyond the scope of this review, only a few key examples are presented. For example, glyburide inhibits ATP-sensitive K⁺ channels downstream of P2X7, resulting in the inhibition of ASC aggregation and IL-1β secretion, independent of its insulinotropic action [379]. Because of their dual functionality, sulfonylureas have been investigated as potential therapies for metabolic inflammatory diseases [380, 381]. β-Hydroxybutyrate (BHB), a ketone body produced during fasting or ketogenic diets, curtails K⁺ efflux, thereby reducing ASC oligomerization and the release of IL-1β and IL-18 [382]. Previous studies have suggested that BHB alleviates gout flares and CAPS symptoms [382]. Recent studies have also explored the anti-inflammatory properties of BHB in neurodegenerative disease models, revealing its potential neuroprotective effects [383–385]. Although regulating Cl⁻ and Na⁺/K⁺ pumps to inhibit NLRP3 activation and IL-1β production is promising [58, 86], developing therapeutics that target major ion channels requires a careful balance between anti-inflammatory efficacy and potential cytotoxicity.

Dimethyl fumarate modulates mtROS, an upstream pathway associated with NLRP3 activation, and it has been clinically approved for psoriasis and multiple sclerosis, in which it reduces NLRP3-mediated neuroinflammation [386, 387]. A recent study assessed its use in autoimmune hepatitis [388]. The monoamine oxidase B inhibitor rasagiline inhibits mtROS production and NLRP3 inflammasome activation [389]. Metformin inhibits NLRP3 inflammasome activation by blocking mtDNA synthesis and the release of cytosolic oxidized mtDNA, thereby improving lung inflammation [390]. Interestingly, nickel–cobalt alloy magnetic nanocrystals can enter cells and downregulate the expression of the lncRNA Neat1—an enhancer of inflammasome assembly—thereby suppressing NLRP3 inflammasome activation [391]. These various approaches—including the modulation of mtROS, prevention of mtDNA release, and regulation of proinflammatory lncRNAs—highlight ongoing efforts to indirectly inhibit NLRP3 and offer potential strategies for treating inflammation-driven diseases. Although several current strategies targeting the upstream and downstream signaling pathways of the NLRP3 inflammasome have demonstrated their potential in ameliorating inflammation and disease severity in preclinical studies using diverse disease models, continued research efforts to evaluate their safety and therapeutic efficacy in clinical settings will ultimately advance the development of effective treatments for NLRP3-driven inflammatory diseases.

### Blockade of IL-1β

Anakinra is a recombinant IL-1 receptor antagonist that binds to IL-1 receptors, blocking both IL-1α and IL-1β [392]. It has shown significant efficacy in reducing CAPS symptoms, gout flares, Still’s disease, rheumatoid arthritis, diabetes mellitus, and heart failure [393–396]. However, its short half-life requires daily injections, which is a drawback for some patients. Rilonacept is a fusion protein that acts as a soluble decoy receptor for IL-1. It sequesters IL-1α and IL-1β, thereby preventing their binding to cellular receptors. The half-life of rilonacept is longer than that of anakinra, thereby permitting weekly dosing [397, 398]. By neutralizing IL-1 before it can trigger downstream inflammation, rilonacept helps reduce disease severity, such as IL-1 receptor antagonist deficiency, CAPS, and recurrent pericarditis, although long-term data on broader indications are still being gathered [399, 400]. Canakinumab is a fully human monoclonal antibody that specifically targets IL-1β [401]. It is highly effective in treating CAPS, significantly improving patients’ quality of life and reducing the risk of amyloidosis [402]. Clinical studies have shown that canakinumab improves other immune-related disorders, such as rheumatoid arthritis, Behcet’s disease-related uveitis, and Schnitzler’s syndrome [403–406]. Furthermore, its success in the Canakinumab Anti-inflammatory Thrombosis Outcome Study trial revealed that IL-1β blockade also lowers cardiovascular event rates in patients with elevated inflammatory markers, highlighting the role of IL-1β in atherogenesis and systemic inflammation [407–409].

The impact of some NLRP3-targeted therapies has been demonstrated in clinical trials, with several promising candidates demonstrating beneficial effects in diverse disease settings [346, 347]. However, translating most experimental findings into widely accepted clinical interventions remains a significant challenge because comprehensive validation of their efficacy, safety, and cost-effectiveness in larger and more diverse patient populations across various stages of multiple NLRP3-related diseases is needed.

## Conclusion

The complicated licensing mechanisms underlying NLRP3 activation, in addition to simple dissection of signals 1 and 2, have been elucidated in this Review. However, many questions remain in terms of understanding the molecular regulation of NLRP3 and its components for the full assembly of the inflammasome complex. K^+^ efflux could be the principal mechanism of inflammasome activation in response to major NLRP3 activators; however, how the intracellular decrease in K^+^ triggers the assembly of the NLRP3 complex is unclear. Additionally, K^+^ efflux may be required for the assembly of other inflammasomes depending on ASC specks, which should be evaluated in future studies.

The identification of numerous molecular partners that function in the positive and negative regulation of the inflammasome will offer promising avenues for future preclinical and clinical research to improve potential therapeutic interventions for various inflammasome-related diseases. Although significant progress has been made in delineating the complex PTMs that govern NLRP3 inflammasome licensing, the distinct roles of each PTM—such as phosphorylation, ubiquitination, acetylation, SUMOylation, citrullination, ISGylation, and palmitoylation—remain insufficiently investigated. Each PTM of an NLRP3 inflammasome component may act as a molecular switch, either independently or collectively, to finely tune NLRP3 priming, activation, and resolution. However, there is a significant lack of knowledge regarding the context-specific functions of PTMs across different tissues, cell types, and disease states.

Addressing these research gaps requires comprehensive mapping of PTM-modifying enzyme expression and activity, which could enable precise modulation of PTMs to regulate inflammasome activity without triggering unintended consequences. Future interdisciplinary studies are needed to explore the impacts of PTMs under diverse physiological and pathological conditions systematically. Additionally, the development of sophisticated in vivo models that accurately mimic PTM-specific perturbations is critical to validate the pathological relevance of NLRP3 inflammasome dysregulation. These efforts will not only advance our understanding of the mechanistic regulation of the inflammasome but also open new therapeutic opportunities through the translation of PTM-related discoveries into clinical applications.

Several unanswered questions and future research directions remain to clarify the intricate and interconnected mechanisms governing the regulation of NLRP3 inflammasome activation and its associated regulatory pathways. For example, what are the specific PTM mechanisms by which positive or negative regulators influence the NLRP3 inflammasome pathway? How do interacting partners modulate the signaling cascades involved in inflammasome priming and assembly? Additionally, the mechanisms by which NLRP3 inflammasome activity is terminated through the restoration of homeostasis remain poorly understood. What roles do key biological responses for intracellular homeostasis, such as the UPR and autophagy, play in controlling NLRP3 inflammasome activation? How do specific PTMs of NLRP3 affect interactions with molecular chaperones, UPR components, and autophagy-related proteins, and what are the consequences of such crosstalk? Furthermore, the interrelationships between PTMs and intracellular signaling networks suggest potential checkpoints for NLRP3 inflammasome licensing and assembly. Could targeting NLRP3 PTM-dependent checkpoints offer future therapeutic modalities for treating NLRP3-related diseases? If so, what are the most promising regulatory points that can selectively inhibit or enhance the interaction activities between NLRP3 and its partners without causing off-target effects on broader cellular functions? A more comprehensive and precise understanding of these mechanisms could facilitate the future clinical application of NLRP3 as an inflammasome regulator in certain inflammasome-related diseases.

Recent structural and functional studies have provided unprecedented insights into the highly dynamic details of the spatial interactions between intracellular organelles and interactive molecules, shedding further light on NLRP3 inflammasome complex formation. Despite these advances, the exact temporal and sequential events involved in NLRP3 inflammasome assembly are obscure in the context of the architectural dynamics of protein complexes and their cellular localization. Moreover, modifications to one spatiotemporal event may influence other processes, potentially leading to unpredictable pathological outcomes. Future studies are needed to elucidate the regulation of structural, functional, and spatiotemporal relationships during the various phases of NLRP3 inflammasome activation. A careful characterization of the spatial and temporal dynamics of PTMs and signaling events will be instrumental in guiding the design of therapeutic strategies to selectively target specific stages of inflammasome activation.

Early-phase studies targeting the NLRP3 inflammasome have highlighted the therapeutic potential of direct NLRP3 inhibitors, upstream and/or downstream modulators, and biological regulators, such as IL-1β inhibitors, in decreasing excessive inflammasome activation. Despite encouraging results in some clinical trials, the translation of experimental findings into clinical applications remains a challenge. Considering the critical role of NLRP3 in the pathogenesis of various inflammatory and autoimmune diseases, future translational research integrating mechanistic insights will be pivotal in the development of novel interventions that precisely target specific phases or key events of NLRP3 inflammasome activation. Continued advancements in these approaches will ultimately refine the clinical use of NLRP3-targeted therapies and improve therapeutic outcomes for various NLRP3-related diseases.
